# Geographical and life-history traits associated with low and high species richness across angiosperm families

**DOI:** 10.3389/fpls.2023.1276727

**Published:** 2023-11-24

**Authors:** Miriam Monserrat Ferrer, Marilyn Vásquez-Cruz, Tania Hernández-Hernández, Sara V. Good

**Affiliations:** ^1^ Departamento de Manejo y Conservación de Recursos Naturales Tropicales, Universidad Autónoma de Yucatán, Mérida Yucatán, Mexico; ^2^ Tecnológico Nacional de México/ITS Irapuato, Guanajuato, Mexico; ^3^ Research and Collections, Desert Botanical Garden, Phoenix, AZ, United States; ^4^ Department of Biology, The University of Winnipeg, Winnipeg, MB, Canada

**Keywords:** diversification rates, depauperons, self-incompatibility, floral traits, life-history traits, whole genome duplication event, growth habit

## Abstract

**Introduction:**

The phenomenal expansion of angiosperms has prompted many investigations into the factors driving their diversification, but there remain significant gaps in our understanding of flowering plant species diversity.

**Methods:**

Using the crown age of families from five studies, we used a maximum likelihood approach to classify families as having poor, predicted or high species richness (SR) using strict consensus criteria. Using these categories, we looked for associations between family SR and i) the presence of an inferred familial ancestral polyploidization event, ii) 23 life history and floral traits compiled from previously published datasets and papers, and iii) sexual system (dioecy) or genetically determined self-incompatibility (SI) mating system using an updated version of our own database and iv) geographic distribution using a new database describing the global distribution of plant species/families across realms and biomes and inferred range.

**Results:**

We find that more than a third of angiosperm families (65%) had predicted SR, a large proportion (30.2%) were species poor, while few (4.8%) had high SR. Families with poor SR were less likely to have undergone an ancestral polyploidization event, exhibited deficits in diverse traits, and were more likely to have unknown breeding systems and to be found in only one or few biomes and realms, especially the Afrotropics or Australasia. On the other hand, families with high SR were more likely to have animal mediated pollination or dispersal, are enriched for epiphytes and taxa with an annual life history, and were more likely to harbour sporophytic SI systems. Mapping the global distribution of georeferenced taxa by their family DR, we find evidence of regions dominated by taxa from lineages with high vs low SR.

**Discussion:**

These results are discussed within the context of the literature describing “depauperons” and the factors contributing to low and high biodiversity in angiosperm clades.

## 1 Introduction

Plants ventured into terrestrial environments for the first time ca. 420 million years before the present (mybp) (Kenrick and Crane, 1997; Magallon et al., 2013; Nie et al., 2020; Su et al., 2021), and proliferated to establish modern land floras. The majority of extant land plant species are angiosperms (90% of species), followed by mosses (ca. 4%), ferns (ca. 3%), and liverworts (ca. 2%), with the remaining species being hornworts, lycophytes, and gymnosperms (Fiz-Palacios et al., 2011; Magallon et al., 2013). Recent studies indicate that the origin of flowering plant orders occurred predominantly in the Early Cretaceous (145–100 mybp) while most extant families radiated during the Paleocene (66–56 mybp) (Magallón et al., 2015; Ramírez-Barahona et al., 2020), although there remains uncertainty in the divergence time of many lineages (Li et al., 2019). Nevertheless, the phenomenal expansion of angiosperm species richness (SR) in that relatively short time span has prompted many investigations into the factors influencing their diversification over geological time (Stanley, 1979; Raup and Sepkoski, 1984; Heard and Hauser, 1995; Nee, 2006; Jablonski, 2007).

Macroevolutionary studies of angiosperm diversification have been dominated by a focus on radiations (Hernández-Hernández, 2019), specifically by studies exploring the possible biotic and abiotic traits that have promoted diversification in lineages that significantly exceed some background level of SR (Magallón and Sanderson, 2001; Magallon et al., 2013; Ferrer et al., 2014; Magallón et al., 2015; Vamosi et al., 2018; Magallón et al., 2019; Soltis et al., 2019; Hernández-Hernández and Wiens, 2020; Ramírez-Barahona et al., 2020). These studies suggest a contextual importance of, for example, innovations in floral morphology and reproduction efficiency that facilitated interactions with pollinators and seed dispersers (Kay et al., 2006a; Sauquet et al., 2017); coevolution with animals (especially pollinators and herbivores; (Xiao et al., 2021); new photosynthetic capabilities (Benton et al., 2022); whole genome duplication (WGD) events (Tank et al., 2015; Clark and Donoghue, 2018); or geographic distribution patterns (Vamosi and Vamosi, 2011). However, significant gaps in our understanding of angiosperm evolution remain; it has been particularly difficult to find universal drivers of increased DR that can explain SR patterns in angiosperms, because traits do not seem to have a consistent effect across clades (Doyle and Donoghue, 1986; Friis et al., 2006; Crepet and Niklas, 2009; Vamosi and Vamosi, 2010; Sauquet and Magallón, 2018; Soltis et al., 2019; Hernández-Hernández and Wiens, 2020; Helmstetter et al., 2023).

A relatively unexplored macroevolutionary pattern ubiquitous along the Tree of Life is the opposite of radiations, the so-called “depauperons”; or lineages showing lower SR than expected (Donoghue and Sanderson, 2015; Caron and Pie, 2022). Depauperons have been recognized since the pre-molecular era, and “living fossils”, or the phenomenon known as “arrested evolution”, are a special case. Living fossils are extant species anatomically similar to a fossil one that occurred early in the history of a lineage (Eldredge and Stanley, 1984). In plants, the genus *Ginkgo* is a textbook example of a living fossil, because it is thought to have appeared at least from the Jurassic period 170 million years (Myr) ago with very little changes in its morphology since (Zhou and Zheng, 2003). Other types of depauperons are the so-known “dead clades walking”, or fossil groups that suffer major drops in their biodiversity at a mass extinction but do not completely disappear (Jablonski, 2007; Barnes et al., 2021). The existence of depauperons represents one of the most interesting puzzles in evolutionary biology, because under normal circumstances, a lineage is expected to either diversify or go extinct, and although theoretical explanations for their existence have been postulated, their drivers remain uncertain (Strathmann and Slatkin, 1983; Barnes et al., 2021). Although overlooked, the study of the evolutionary mechanisms sustaining depauperons in contrast to those driving radiations or angiosperm “success stories” will likely illuminate explanations of SR patterns across flowering plants (Caron and Pie, 2022).

To explore SR patterns in angiosperms from a depauperons perspective, our study aims at identifying plant traits and geographic distribution patterns associated with angiosperm families that have unusually low SR. For this purpose, we followed a consensus maximum-likelihood (ML)-based approach to model the diversification rate process in angiosperm families, and identified families with significantly higher or lower DR/SR than expected by using five relevant calibrated angiosperm reconstruction datasets that vary in the sampling, calibration, and statistical approach. Then, using only those families categorized as having low, expected, or high SR across all five studies (strict consensus), we assessed if family SR was associated with traits that have been recurrently reported as having an impact on angiosperm DR: (i) the presence of an ancestral WGD event; (ii) 23 intrinsic traits from published studies including traits related to flowering morphology, mode of pollination or fruit dispersal, and growth habit; (iii) variation in sexual system, specifically the presence of dioecy, or self-incompatibility (SI) mating system; and/or (iv) the geographical distribution of families. Our traits dataset compiled an updated version of a previous one (Ferrer and Good, 2012) with previous datasets (Hernández-Hernández and Wiens, 2020), and we included a curated version of georeference dataset for all angiosperm families studied. Our results reveal important patterns in the distribution of depauperons, which, although are not a constitutive component of floras around the world, compose ~30% of angiosperm diversity at a family level, and include several vulnerable taxa that are highly endemic. Our results are discussed within the context of the literature describing “depauperons” (sensu Donoghue and Sanderson, 2015), and the wealth of literature underscoring the importance of breeding systems and animal–plant interactions in biodiversity conservation.

## 2 Materials and methods

### 2.1 Crown age and species richness of angiosperm families

Since the taxonomic delineation of families has changed since the oldest study we included (Wikström et al., 2001), and families have been split, merged, etc. over time, the family names and relationship included in this study reflect the most recent classification recognized by the APGIV (The Angiosperm Phylogeny Group et al., 2016) (**Table 1**). The SR for 432 of the 434 families recognized by the APG IV was obtained from the angiosperm phylogeny site (Stevens, 2021); when the number of species in a family was presented as a range, the mean was used as the SR. The crown age of families was obtained from five calibrated angiosperm phylogenies: Wikström et al. (2001); Bell et al. (2010) (exponential), Hernández-Hernández and Wiens (2020); Li et al. (2019), and Ramírez-Barahona et al. (2020) (RC_complete_MCCv_2.tre as recommended by the authors). The crown age of families not included in a study was set to that of its’ sister clade according to the APGIV, or to the stem age in the case of monotypic families or those in which only one species has been genotyped to represent the family (Stevens, 2021). The dataset names were abbreviated throughout the paper using the last name of the first author as follows: (1) Wikström (Wikström et al., 2001), (2) Bell (Bell et al., 2010), (3) Hernández-Hernández (Hernández-Hernández and Wiens, 2020), (4) Li (Li et al., 2019), and (5) Ramírez-Barahona (Ramírez-Barahona et al., 2020).

**Table 1:** Genes, number of families, outgroup taxa, and fossils included in the five calibrated phylogenies employed in this study: Wikström et al. (2001); Bell et al. (2010); Hernández-Hernández and Wiens (2020); Li et al. (2019), and Ramírez-Barahona et al. (2020).

Phylogeny	Genes	No. of families			Outgroup taxa	Fossils
		Original work	APGIV	Inferred ages		
**Wikström**	*rbcl, atpB, 18S rDNA*	340	369	65	7	1
**Bell**	*rbcl, atpB, 18S rDNA*	335	295	137	7	36
**Hernández-Hernández**	*rbcl, atpB, matK, 18S, 26S rDNA*	363	402	30	7	137
**Li**	*80 (plastid)*	353	353	79	163	62
**Ramírez-Barahona**	*rbcl, atpB, matK, ndhF, 18S, 26S, 5.8S, rDNA*	435	402	30	7	238

Given the changes in the identity of angiosperm phylogeny group (APG) recognized families since 2001, the families considered in the original work were lifted over to APGIV, and the age of missing sister clades inferred following APGIV.

### 2.2 Shifts on the diversification rate of angiosperms

Since diversification patterns can change through global events in different geological epochs, we divided the data into time intervals during which the diversification rate followed a uniform distribution (following Ferrer et al., 2014). To identify significant shifts in DR, the original trees from each dataset were trimmed to exclude outgroups and linearized to be ultrametric with respect to time (if not already ultrametric) using an R script (available from https://hcliedtke.github.io/R-scrapheap/be_ultrametric.html). Subsequently, potential shifts in the rate of speciation or extinction were modeled using reversible jump MCMC as implemented in the program TESS (Höhna et al., 2016) (**Supplementary Data File 1**); shift points were inferred when 2lnBayesFactor ≥ 2 and the median age of the peak was used to divide the data into periods with approximately uniform DR. Once divided, we employed an MLE approach (below) to estimate the DR parameters and their 95% CI within each period.

### 2.3 Estimates of the speciation, extinction, and diversification rate of angiosperm families

We used the SR/clade age data from each of the five datasets to estimate the average absolute diversification rate of angiosperm families using a birth–death model. For all families originating within each geological interval, MLEs of ${\hat{r}}_{\lambda -\mu },\;\lambda ,\;\mu ,\;\varepsilon$ and their 95% CIs were estimated from the function β (see equation 2b, from Magallón and Sanderson, 2001) as:


$$\beta =\frac{\lambda \left[e^{{\left(\lambda -\mu \right)}^{t}}-1\right]}{\lambda \left[\lambda e^{{\left(\lambda -\mu \right)}^{t}}-\varepsilon \right]}$$

and then the MLE of ${\hat{r}}_{\lambda -\mu },\;\lambda ,\;\mu ,\;\varepsilon$ obtained using the following likelihood equation (Bokma, 2003).


$$L(\lambda -\mu )=lnL\;\sum_{i}^{k}\ln(1-\beta_{i})+\;\sum_{i}^{k}[(n-1)ln\beta_{i}]$$

For each time interval per study, the likelihood of the model (pSp) was estimated using diversification (*r*) rate values from 0.0001 to 0.2, and speciation rates (λ) from 0.1 to 100 species (per million years), in increments of 0.0002 for *r* and of 0.01 for λ using the divergence age ( $t_{i}$ ) and SR of each family ( $n_{i}$ ) following Bokma (2003) with the aid of Maple (see **Supplementary Data File 1**).

Next, two sets of CIs were estimated: CIs for the MLEs of ${\hat{r}}_{\lambda -\mu },\;\lambda ,\;\text{and }\mu$ , were estimated from the likelihood surface and a second set of CIs were obtained to account for error in the expected SR of families under a stochastic diversification process. In the first case, we used the fact that when estimating related parameters via ML (i.e., $\hat{r}=\;\lambda -\mu$ ), approximate CIs are given by ±1 unit of the likelihood surface (Meeker and Escobar, 1995). The ML surface generated following Eq. (2) was explored to consider all models within one likelihood unit of the best model, and then the 95% CIs were taken to be the lowest and highest values of λ and *r*, and by deduction, μ . Secondly, CIs associated with the number of species per clade under the birth–death model were obtained by solving equations 10a and 10b from Magallón and Sanderson (2001) using the following equations:


$$k_{(upper)t=\;\frac{\ln\left(0.025\right)}{\ln\left(\beta \right)}+1}$$

$$k_{(lower)t=\;\frac{\text{ln}\left(0.975\right)}{\text{ln}\left(\beta \right)}+1}$$

where α and β were obtained by solving α_t_ and β_t_ as given in Magallón and Sanderson (2001) from equation 8.47 of Bailey (1964), in which ${\hat{r}}_{\lambda -\mu }\;\;\text{and }\epsilon$ were taken as the value of the net diversification rate and extinction fraction that maximized the likelihood given in equation 2, where *t* was the age of divergence of the family.

### 2.4 Assigning families as having rich, predicted, or poor species richness

Using the maximum width of the CI, families were classified as having low, predicted, or high SR if the number of species in the family fell below, within, or above the maximum or minimum CI, respectively. To assess whether family classification was independent of the time period in which the family originated, a χ^2^ with Haberman residual analysis was performed (Haberman, 1973). Finally, if families were identified as having low, predicted, or high SR based on strict (5/5) consensus across datasets, they were classified as having “poor”, “expected”, or “high” SR overall, while families lacking consistency across datasets were labeled as “undefined”.

### 2.5 Traits associated with families differing in species richness

We tested whether the presence of a WGD event within the evolutionary history (crown or stem age) of a family based on the data from Landis et al. (2018), and/or the presence of fleshy fruits or floral symmetry classification, following Vamosi and Vamosi (2011) and Reyes et al. (2016), respectively, were associated with SR category. A χ^2^ contingency analysis followed by a Haberman residual analysis (Haberman, 1973) was used to test for significance. Next, we looked for associations between family SR category and data on 21 traits compiled by Hernández-Hernández and Wiens (2020). The traits were divided into those related to the proportion of species in a family with a given: mode of dispersal (biotic, insect, vertebrate, water, or wind), fertilization (via biotic pollinator, insects, vertebrate pollinator, water, or wind), growth form (climbing, herbaceous, shrub, or tree), habitat (aquatic, epiphytic, or terrestrial), mean seed weight, or the proportion presenting with an annual life-history form, nectar spurs, polyploidy, or dioecy. Kruskal–Wallis tests followed by Dunn’s *post-hoc* comparisons were used to test if the distribution of trait values between SR categories differed using a global FDR of 0.1.

### 2.6 Association of mating and sexual systems among families differing in species richness

#### 2.6.1 Presence of SI

We used an updated version of the database from Ferrer and Good (2012) describing evidence for the presence of a self-sterility (SS), self-compatibility (SC), or one or more types of genetically controlled self-incompatibility (SI) systems in angiosperm families to test if there was an association between mating systems and family SR category. Families were classified as self-compatible when >80% of the species examined in a family exhibited SC, as SC-SI when >20% but<80% of species were classified as SI or SS, or as self-incompatible when >80% of the species in a family were classified as having SI or SS.

#### 2.6.2 Presence of dioecy

Information about sexual system (dioecy) was included using the proportion of dioecious taxa obtained from Renner (2014) and Hernández-Hernández and Wiens (2020): only families in which all of the species exhibited dioecy were considered to be dioecious clades.

#### 2.6.3 Type of SI

To assess if there are differences in the type of SI across families, we updated the database of Ferrer and Good (2012), to assign families with SI as having GSI (gametophytic self-incompatibility), SSI (sporophytic self-incompatibility), LAS (late-acting self-incompatibility), HET (heterostyly), HET + SI, or poly (multiple forms of SI in the same family), or as being unclassified (when there was evidence of self-sterility but unknown SI system). Inclusion criteria for designating a species as having genetically controlled SI required evidence from (unsuccessful) cross-pollinations between individuals putatively harboring the same S-genotype, microscopic analyses of pollen tube arrest in the stigma (SSI), pistil (GSI), or ovary (LAS), and/or expression of known S-alleles. In total, data on 6,441 species were extracted from manuscripts published between 1940 and 2022, and data on the type of SI in the 248 angiosperm families with an SR category based on strict consensus across studies included in the analyses. After removing 88 families with unknown mating systems, 111 families with some form of SI or SS, 38 families with SC, and 11 families with dioecious taxa were identified.

### 2.7 Geographic distribution of families differing in species richness

To evaluate the geographic distribution and range of families, we used the Global Biodiversity Information Facility database (GBIF, gbif.org). Only records from preserved specimens and literature with georeferenced coordinates were downloaded. All family names were corroborated and standardized according to The Plant List (The Plant List, available now http://www.worldfloraonline.org/) using the taxonstand package in R software (Cayuela et al., 2012). We removed all records lacking full genus and species names, and eliminated species records with duplicated georeferenced data, and those from common crops, cultivated specimens, botanical gardens, or from greenhouses or markets. The geographical distribution of taxa was finally corroborated manually at the family level, based on information for each family in the Kubitzki system (Kubitzki, 1990 onward), and online platforms (https://www.tropicos.org/home, http://www.mobot.org/MOBOT/research/APweb/; http://www.worldfloraonline.org/). This curated georeferenced database was used to map the mean DR of taxa belonging to families with high vs. poor SR. To do this, we first calculated the mean DR of the 75 families with poor SR and the 12 families with high SR (**Supplementary Data File 4**). Next, we divided the globe into grids consisting of 1 degree squared. Then, within each grid, the average DR of all the families to which each georeferenced data point (taxa) belonged was calculated and plotted. Thus, the color of each pixel on the map represents the global mean DR of the families represented by the taxa georeferenced to that location.

### 2.8 Distribution of families with different SR categories in global biomes and realms

The same curated databases were used to score the presence of families in global biomes and realms, as defined by Olson and Dinerstein (1998). We used the bioregionalization categories from the Terrestrial Ecoregions of the World (TEOW), with a subdivision of the terrestrial world into 14 biomes and eight biogeographic realms. For each family, we counted the percentage of records falling within each realm and biome; thus, the maps generated from this analysis depict the density of taxa from families with low, expected, or high SSR across the globe. Next, to analyze the distributional data using contingency analysis, the distributional range of families with poor, predicted, or high SR were categorized as highly localized, localized, widespread, and cosmopolitan by visual arbitrary assessments with the following criteria: highly localized (when all records were concentrated in a single country or small region), localized (when all records were concentrated in a larger area within a continent), widespread (when the distribution of records span more than one continent), and cosmopolitan (when the records were distributed in several continents). In addition, and by visual assessment, the distributional pattern for families was categorized as disjunct (distributed in clusters but in two separate continents, or clusters separated by a considerable large area without continental contiguity) or continuous (no disjunctions).

## 3 Results

### 3.1 Crown age and species richness of angiosperm families

The five studies employed overlapping, but not identical, sets of molecular markers, primarily derived from the chloroplast genome, but differed markedly in the number of fossils used to calibrate the phylogeny and the methods used to calibrate the phylogeny (**Table 1**). Of the 432 angiosperm families, 235 were included in all five studies, and crown ages were available for 295 (Bell) to 402 (Hernández-Hernández and Wiens, 2020, and (Ramírez-Barahona et al., 2020) families recognized by APGIV, such that ages were inferred using the (missing) family’s age for 137 to 30 families (**Table 1**).

### 3.2 Shifts on the diversification rate of angiosperms

**Figure 1:**
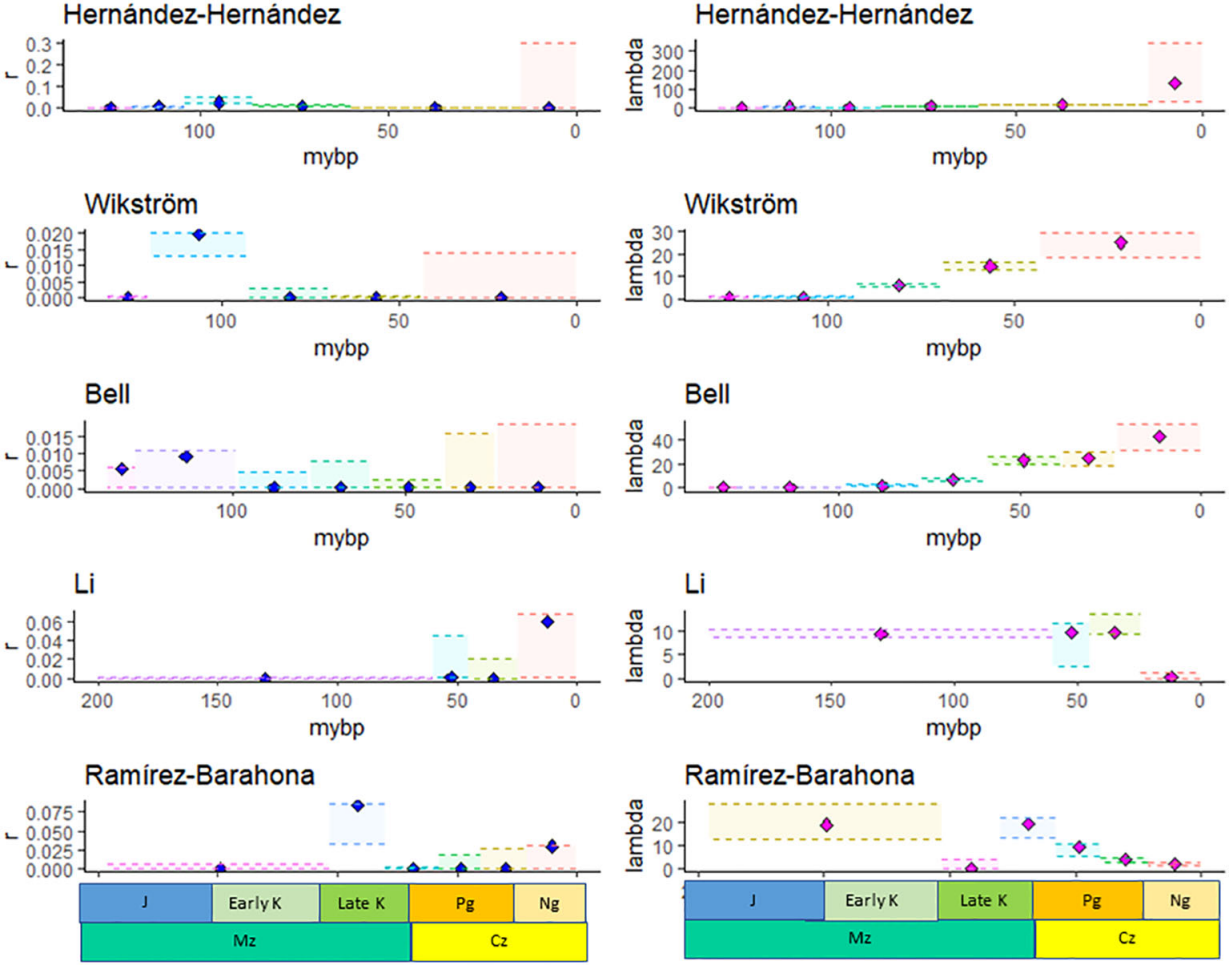
The maximum likelihood estimate, MLE (diamonds), and minimum and maximum confidence interval for *the net diversification rate*, r*, and the speciation rate*, λ*, estimated using the method of* Bokma (2003) *based on the species richness of families given by APG IV and the crown age of families estimated by* Hernández-Hernández and Wiens (2020); Wikström et al. (2001); Bell et al. (2010); Li et al. (2019) *, and* Ramírez-Barahona et al. (2020).

Bayesian analysis identified shifts in the DR of angiosperm families in all of the ultrametric calibrated phylogenies, and inferred either four (Li), five (Wikström), six (Hernández-Hernández and Ramírez-Barahona), or seven (Bell) shifts in DR (**Supplementary Data File 2**, **Table S1**; **Figure 1**).

### 3.3 Estimates of the speciation, extinction, and diversification rate of angiosperm families

The MLE of the DR in most time intervals and for most phylogenies was low (*r* ~ 0.0001, **Supplementary Data File 2**, **Table S2**), although, in some cases, the CIs were high (e.g., Bell). In four of the five studies, *r* was highest for families that originated in the Albian-Turonian (113–100.5 mybp), while in the Li et al. study, *r* was the highest for families originating during the Neogene (**Figure 1**). The speciation rate (λ) estimated from the Wikström, Bell, and Hernández-Hernández datasets increased towards the present and ranged from 25.1 to 132.60 during the Cenozoic (**Figure 1**). On the other hand, λ (**Figure 1**) and μ decreased towards the present based on the Li and Ramírez-Barahona datasets (**Supplementary Data File 2**, **Table S2**).

The MLEs of the DR parameters based on 432 angiosperm families were similar to those estimated from the original number of families included in each phylogeny (**Supplementary Data File 2**, **Tables S2, S3**, respectively), although the diversification and speciation rates were higher in the original phylogeny due to the lack of inclusion of families with low SR.

### 3.4 Assigning families as having rich, predicted or poor species richness

**Figure 2:**
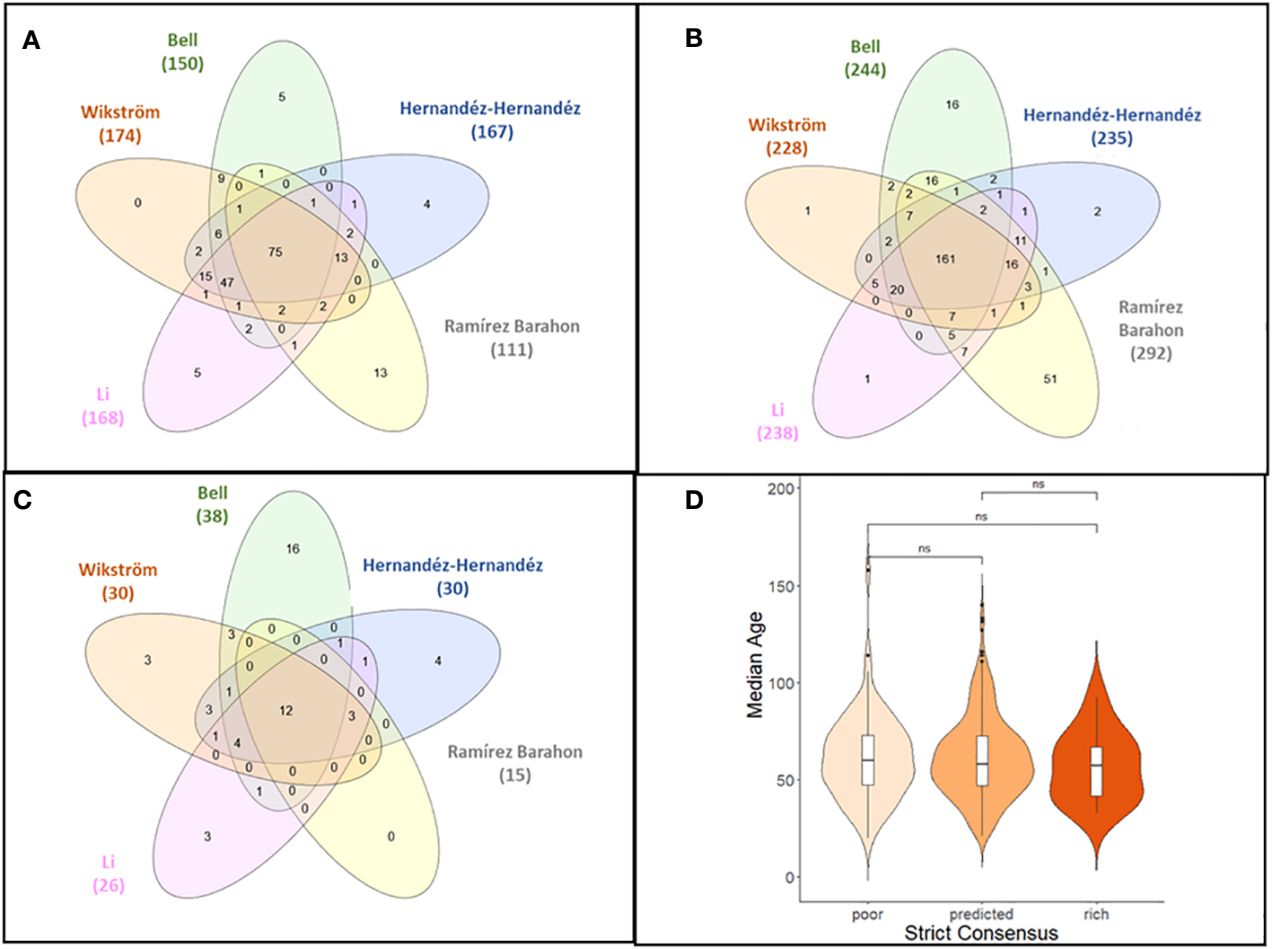
**(A–C)** Venn diagrams showing the levels of agreement between classification of angiosperm families with low **(A)**, expected **(B)**, or high **(C)** levels of species richness as inferred from five datasets. The strict consensus is given in the center. **(D)** Violin plot of the median age of angiosperm families across the five phylogenetic studies employed in the analysis by family classification category (poor, predicted, or rich) based on strict consensus criterion. The distribution of the median crown ages was not significantly different for families with poor, predicted, or high SR using the Kolmogorov–Smirnov test.

Using strict consensus criteria across the five studies, 248 angiosperm families were classified as having either predicted SR (161 families, 65% representing 73,803 total species), poor SR (75 families 30.2%, respectively representing 196 species), or high SR (12 families, 4.8%, representing 133,802 total species) (**Figures 2A–C**; **Supplementary Data File 4**). Families with poor SR typically had<10 species, while families with high SR had >4,000 species (**Supplementary Data File 2**, **Table S4**). Using only those families that had a crown age estimated in the original phylogeny, the trend was similar, though the number of families in the poor category was lower since families with low SR were more likely to be not included in the phylogenetic reconstructions (**Supplementary Data File 2**, **Table S4**).

**Figure 3:**
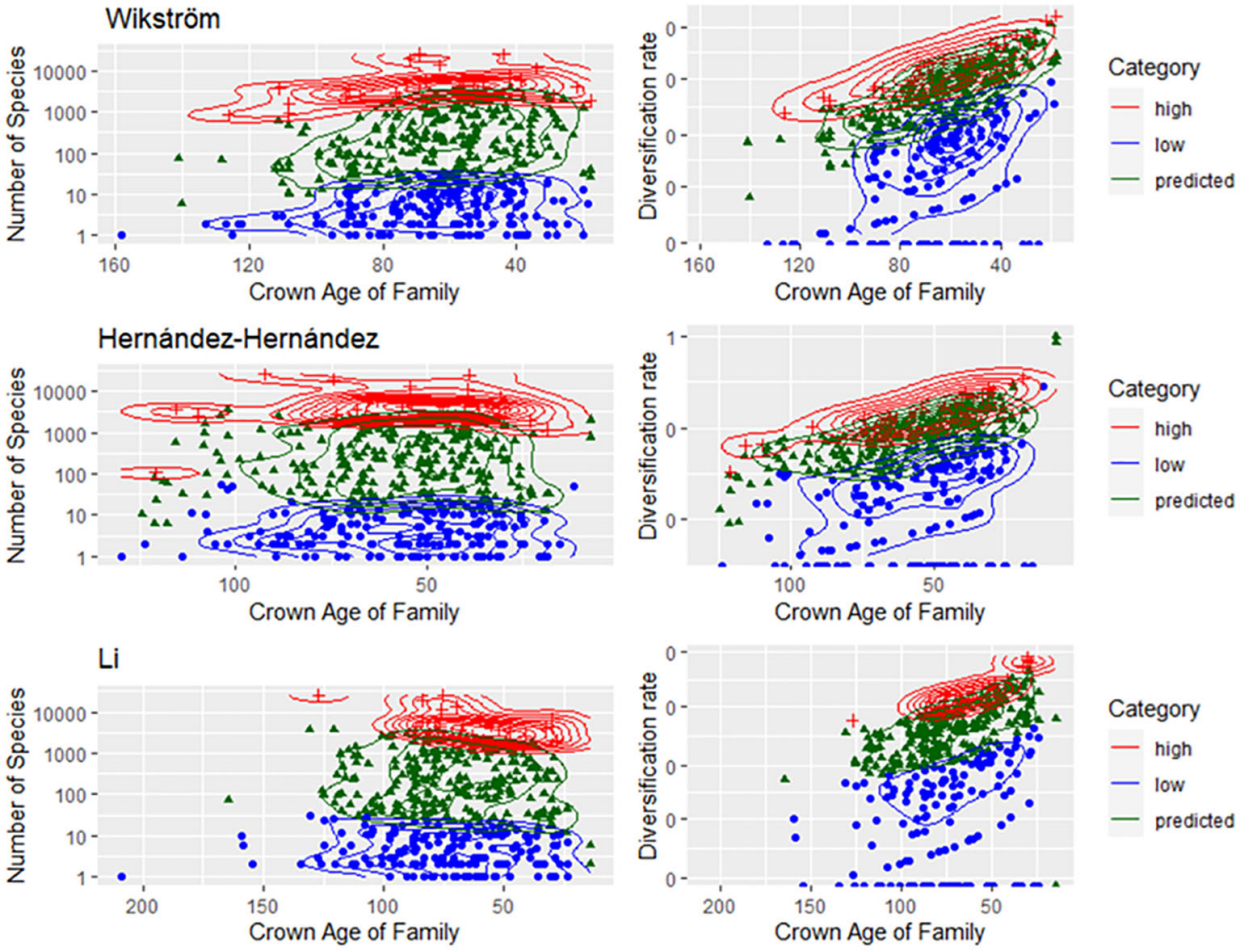
Families categorized as having high, predicted, or low SR (left panel) or DR (right panel) by family crown age based on the datasets from Wikström et al. (2001); Hernández-Hernández and Wiens (2020), and Li et al. (2019).

**Figure 4:**
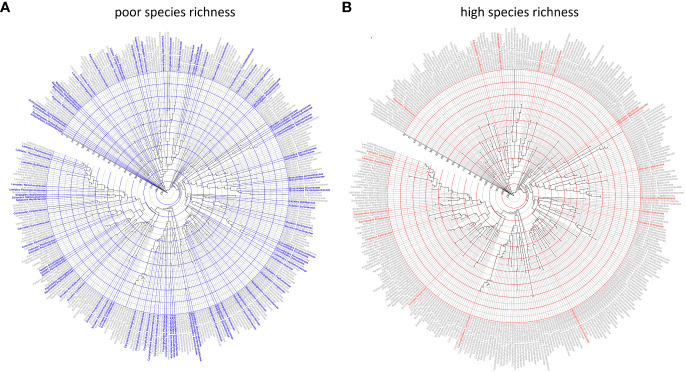
Ultrametric molecular phylogeny of angiosperm families based on Hernández-Hernández and Wiens (2020). The family name and external branches are colored by the inferred species richness status, estimated using the MLE modeling described in the methods. The root of the phylogeny (Amborellaceae) is 132 mybp; concentric circles are placed every 14 mybp to the present. **(A)** Families with poor species richness based on majority consensus (4/5 studies in agreement) criterion. **(B)** Families with high species richness based on majority consensus (4/5 studies in agreement) criterion. Solid lines indicate families for which the SR category was declared by strict consensus, and dotted lines denote families for which the SR category was declared based on majority consensus.

There was no evidence that family SR category was associated with the crown age of the family (**Figure 2D**). Based on the independence test, only the Bell and Ramírez-Barahona datasets had more families than expected that had high (99–128 mybp period) or low (103.1–196 mybp period) SR within a given geologic interval (**Supplementary Data File 2**, **Tables S5, S6**). For the remaining studies, the inferred SR of a family was independent of the period in which the family originated, and was predominantly so for the Bell and Ramírez-Barahona datasets (**Supplementary Data File 2**, **Table S5, S6**). As expected, DRs increased towards the present (**Figure 3**, right panels), but the number of families with low, predicted, or high SR appeared evenly distributed over time when plotted on a log scale (**Figure 3**, left panels). The Bell dataset showed the same pattern, while in the Ramírez-Barahona dataset, the DR of families tended to increase towards the present, but the speciation rate declined (**Supplementary Data File 3**, **Figure S1**). In agreement with this, the Kolmogorov–Smirnov test found no difference in the distribution of median ages of families with poor, predicted, or high SR based on strict consensus criterion (*D* = 0.8, *p* > 0.1; **Figure 2D**). Furthermore, there is no evidence of phylogenetic clustering of families with poor or high SR (**Figure 4**).

### 3.5 Traits associated with families having different species richness

Given that previous studies have shown that lineages experiencing an ancestral WGD event are more likely to exhibit sustained and elevated DRs, we performed an independence test of the relationship between SR category and the presence of one or more WGD events near the crown age of a family, using the inferred WGD events from Landis et al. (2018). This indicated that families with high SR were more likely to have more than two WGD events, while families with poor SR were more likely to have no WGD events (χ^2^ = 73.65, *p*< 0.0001) (**Table 2**).

**Table 2:** Frequency of families with poor, predicted, rich, or mixed species richness based on strict consensus tabulated by the absence or presence of a WGD event (data from Landis et al., 2018).

WGD	Poor	Predicted	High	Total
**None**	71	119	**2**	192
**One**	**3**	39	5	47
**> Two**	1	3	**5**	9
**Total**	75	161	12	248

Results are presented for the strict consensus categories across five datasets from angiosperm phylogenies (χ^2 =^ 73.65, p< 0.0001). Frequencies in blue font are those displaying fewer families than the expected, while those in red are those with more families than expected according to Haberman’s residual test (Haberman, 1973).

**Figure 5:**
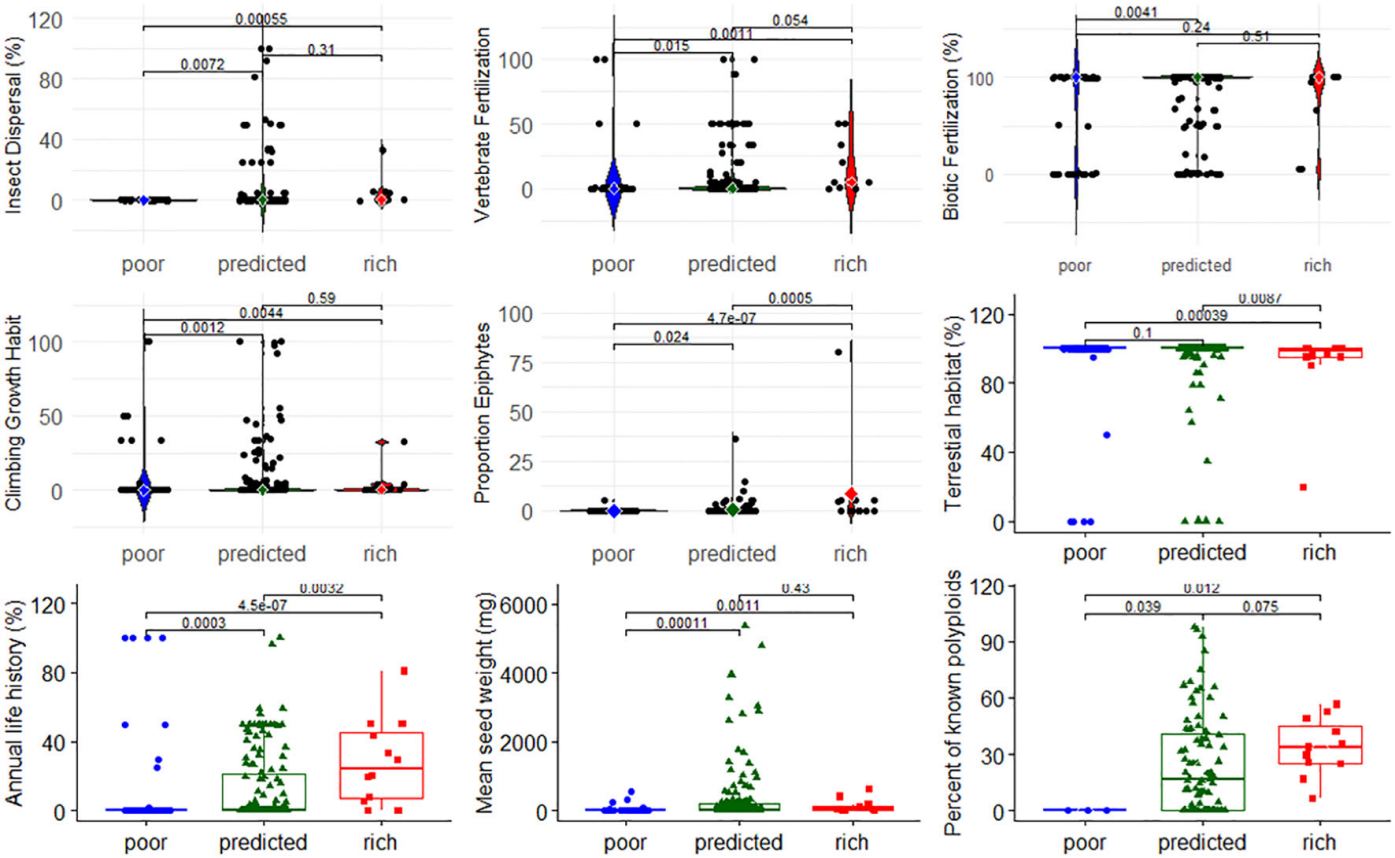
Distribution of nine traits across families with poor, predicted, or high species richness based on strict consensus. Differences in the proportion of species per family between SR categories were analyzed using a Kruskal–Wallis, non-parametric rank test, at an FDR = 0.1. The remaining 11 traits with non-significant differences among SR categories are shown in the **Supplementary Data File 3** (**Figure S2**).

Next, we investigated whether angiosperm family SR category was associated with specific traits. We find that SR classification was associated with floral symmetry patterns (χ^2^ = 35.91, *p* = 0.0047), and families with poor SR were less likely to have zygomorphic flowers and more likely to have spiral floral symmetry (**Data File 2**, **Table S8**). Using the data on the distribution of 20 angiosperm traits from Hernández-Hernández and Wiens (2020) and a global FDR = 0.1 (*p*-value threshold = 0.005), we found that 8 of the 20 traits exhibited a significant difference in trait value between family SR category, while one showed a trend (**Figure 5**). Families with poor SR were less likely to have insect pollen dispersal, vertebrate fertilization, and epiphytic taxa; reside in terrestrial habitats; or have an annual life history relative to families with high SR, and were less likely to have a climbing growth habit and an annual life history, and had lower mean seed weight compared to families with predicted SR. There was a clear but non-significant trend that the percent of polyploid species in families increased with SR category (**Figure 5**). The remaining 11 traits exhibited no significant differences among family SR class (**Supplementary Data File 3**, **Figure S2**), and there was no association of family SR with the presence of fleshy fruits (χ^2^ = 0.081, P = 0.667, **Data File 2**, **Table S7**).

### 3.6 Association of different mating and sexual systems among angiosperm families with different species richness

Comparison of the sexual and mating systems across the 248 angiosperm families revealed a strong association between family SR and mating and sexual system (χ^2^ = 101.72, *p*< 0.0001). Families with poor SR are more likely to be composed of exclusively dioecious taxa and (for those families that are not dioecious) more likely to have unknown mating systems, while families with predicted SR are less likely to be dioecious (**Table 3**). Interestingly, families with high SR are more likely to harbor intermediate numbers of taxa with SI systems (SC-SI, **Table 3**), and are more likely to have SSI (**Supplementary Data File 2**, **Table S9**).

**Table 3:** Frequency of families with poor, predicted, rich, or mixed species richness based on strict consensus tabulated by the sexual system (data from Renner, 2014) and mating system type (data from Ferrer and Good, 2012).

SI status	Poor	Predicted	Rich	Total
**Dioecious**	10	2	0	11
**SC**	8	35	0	47
**SC-SI**	4	57	12	70
**SI**	3	30	0	32
**Unknown**	50	37	0	88
**Total**	75	161	12	248

Results are presented for the strict consensus categories across five datasets from angiosperm phylogenies (χ^2 =^ 104.62, p< 0.0001). Frequencies in blue font are those displaying less families than the expected, while those in red are those with more families than expected according to Haberman’s residual test (Haberman, 1973).

SC, Self-compatible (>80% of species within the family are self-compatible); SI, Self-incompatible (>80% of species within the family are self-incompatible); and SC-SI, (<80% and >20% of species within the family are self-compatible).

### 3.7 Geographic distribution of families differing in species richness

**Figure 6:**
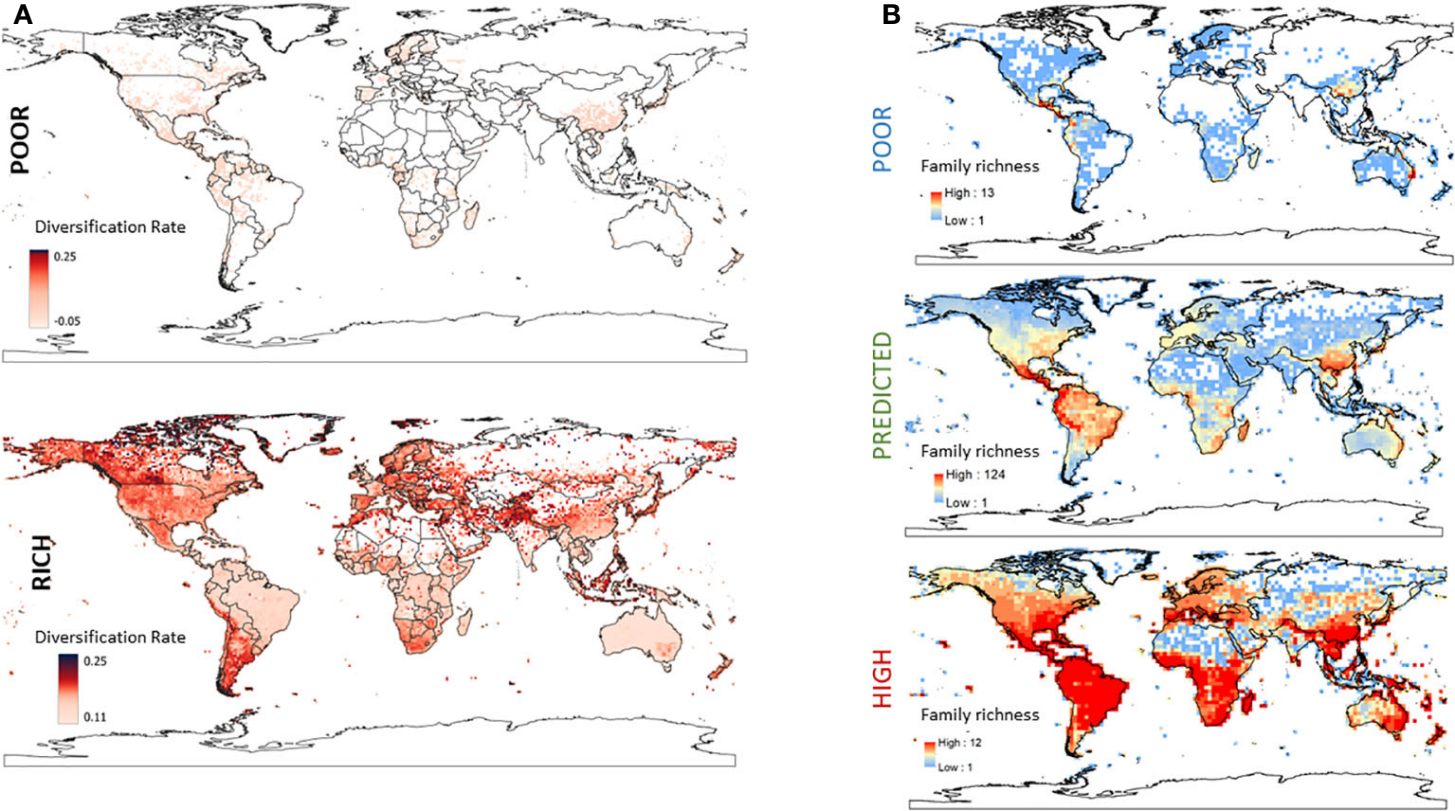
**(A)** The mean diversification rate (DR) of georeferenced taxa from families with poor SR (top) or high SR (bottom). The colour of each pixel on the map represents the global mean DR of the families represented by all taxa georeferenced to that location. The corresponding figure for families with predicted SR is in the supplementary data (**Data File 3**, **Figure S3**). **(B)** Global distribution of taxa in families with poor, predicted and high species richness. The geographic distribution of the SR of taxa in families classified as having poor (top left), predicted (middle left) or high (bottom left) SR. Intensity of red scale indicates the number of taxa/family in that geographic region.

We plotted the mean DR of all georeferenced data for families with poor SR (**Figure 6A**, top) and high SR (**Figure 6A**, bottom). The mean DR of georeferenced taxa from families with poor SR varied from −0.04 to +0.04 and families with poor SR appear to be non-randomly distributed in the globe. Keeping in mind that the GBIF database does not equally sample the world’s flora, families with poor SR are more represented in Japan and Southern China, parts of Indonesia, Western and Southern Africa, Northern Europe and Spain, and throughout MesoAmerica and North America. On the other hand, the mean DR of georeferenced taxa from families with high SR ranged from 0.11 to 0.249. Families with high SR were dominant in grasslands (particularly in Central Canada and Patagonia) consistent with two families in the Poales having high SR (the Poaceae and Cyperaceae), as well as many mountainous regions, including the Rockies, Andes, Atlas, Himalayas, and others mountain ranges.

Lastly, we used independence tests to assess the association between family SR category and four metrics of geographic distribution: distributional range and pattern, presence in biome, and realm. There was a strong association between family SR and distributional range (χ^2^ = 238.67, *p*< 0.0001), but a modest one for distribution pattern (χ^2^ = 6.47, *p* = 0.0393); families with poor SR were more likely to exhibit highly localized or localized ranges, and were less likely to be widespread, while families with predicted SR exhibited the opposite pattern and those with high SR were more likely to have a cosmopolitan distribution (**Data File 2**, **Tables S10, S11**, **Data File 3**, **Figure S3**). Similarly, families with poor SR were more likely to be present in a single realm, while families with predicted SR were more likely to found in five to seven (of the eight) realms, and families with high SR were found in all eight realms (**Data File 2**, **Table S12**, **Figure 6B**). There was a non-random association of family SR category and realm presence (χ^2^ = 53.97, *p*< 0.0001, **Data File 2**, **Tables S13, S14**), and families with poor SR were more likely to be located in the Afrotropics and Australasia while families with predicted SR were less likely to be found in Australasia (**Figure 6B**, **Data File 2**, **Tables S13, S14**). Turning to biome number, not surprisingly, families with poor SR were also more likely to be present in 1 or 2–4 biomes while predicted and high SR families were more likely to be found in 11–13 (predicted SR families) or all 14 biomes (high SR families) (**Data File 2**, **Table S15–S17**).

## 4 Discussion

The study of depauperons, or lineages having a lower-than-expected SR, has received much less attention than the opposite pattern, the evolutionary radiations (Donoghue and Sanderson, 2015; Caron and Pie, 2022). This is partly because monotypic and/or low species-rich families have not been consistently included in molecular phylogenetic studies owing to the difficulty of obtaining specimens. However, much progress has been made to include them in the Tree of life (To), and ongoing efforts to sequence under-represented taxa in the angiosperm tree, such as the oneKB initiative (Leebens-Mack et al., 2019), make the study of depauperons feasible. Another reason why there has been less attention to depauperons lies in the difficulties to assess whether plant families have unusually low SR due to the complexity of diversification rate models, the existence of broad shifts in the inferred DR over evolutionary time, and the wide variation in the estimated crown and stem age of taxa across studies that employ different markers, statistical methods, and fossil data (Sauquet and Magallón, 2018; Benton et al., 2022; Helmstetter et al., 2023). In this study, we aimed to account for these limitations by (1) estimating the age of significant shifts in the DR rate of angiosperm families using Bayesian methods in each of five large phylogenetic studies and then dividing the data into geological intervals within which DRs could be expected to be uniform and then (2) employing the approach of Bokma (2003) to obtain MLEs of the DR parameters and their 95% CIs within each geological interval including two factors contributing to measurement uncertainty, (3) using families as the taxonomic unit of choice since plant families continue to be robust to insights from molecular phylogenetic analysis (Soltis et al., 2005), and (4) using strict consensus criteria to categorize family SR, so that our inferences regarding trait evolution were robust to the uncertainties in the inferred crown age of families. In contrast to many other studies that examine the relationship between DR/SR and other variables, our approach is not dependent on the topology of any phylogeny.

Based on the 248 families that met the strict consensus criterion, we find that the majority of them have an expected SR (65%), while interestingly, 30.2% have lower SR than expected, while only 4.8% have an SR that is higher than expected. This points to important inferences from this analysis, namely, that (i) almost 1/3 of angiosperm families have low SR and (ii) despite finding strong evidence for shifts in the mean rate of diversification of angiosperms over geological time, angiosperm family SR is not strongly correlated with clade age, as previously observed in both plants (Magallón and Sanderson, 2001; Vamosi and Vamosi, 2011) and animal lineages (Rabosky et al., 2012).

### 4.1 Shifts in angiosperm diversification rate

All five of the phylogenetic studies employed in our analyses showed evidence of a shift in the rate of extinction or speciation between 90 and 110 mybp, corresponding to the Albian-Turonian, and usually an increase in the rate of speciation between 50 and 70 mybp, corresponding to the Cretaceous/Paleocene (K/T) boundary, recently called the Angiosperm Terrestrial Revolution (Benton et al., 2022). These results corroborate other studies indicating that angiosperm families radiated mainly during the early Cretaceous and the Cenozoic (Magallón et al., 2015; Sauquet and Magallón, 2018; Li et al., 2019; Onstein, 2020; Ramírez-Barahona et al., 2020), while the diversification of orders (or stem ages) of angiosperms occurred during the Albian-Turonian (Magallón et al., 2015; Ramírez-Barahona et al., 2020). Fossil data show that ~80% of plant species exhibited a sudden extinction at the K/T boundary, which was paralleled by the extinction of many insect species (Johnson et al., 1989; Johnson, 2002; Labandeira et al., 2002). Subsequently, DRs remained low for an estimated 10 my (Wing and Boucher, 1998), but were followed by a radiation during the Paleocene and Eocene (Niklas, 1997; Feild et al., 2004; Soltis et al., 2005).

Cycles in which stasis and sporadic bursts of speciation intermingle are characteristic of angiosperm evolution and have been proposed to be related to the presence of “Greenhouse” and “Icehouse” periods that characterized the earth’s global climate before and after the Tertiary period (Willis et al., 2004). During the Paleocene–Eocene transition, high global temperatures reached a maximum at approximately 55.8 mybp (Wolfe, 1978; Zachos et al., 2001), which coincided with an increase in low-latitude palynofloral diversity (Wing and Currano, 2013; Landis et al., 2018), including the diversification and expansion of rainforests worldwide (Morley, 2000; Carvalho et al., 2021; Jaramillo, 2023), and the diversification of biotic taxa that could serve as both pollinators and seed dispersers (Van Der Kooi and Ollerton, 2020; Benton et al., 2022). There is a growing consensus that the increased rate of speciation of angiosperm families at the Paleocene–Eocene boundary may be associated with (1) a decrease in the number of angiosperm species and therefore an opening of niches after the K/T event, (2) climatic conditions favoring speciation in the early Tertiary to Oligocene, and (3) the diversification of biotic pollinators and seed dispersers that both facilitated and reinforced the evolution of intrinsic plant traits that allowed species to invade new habitats and maintain high genetic diversity while also providing pollinators and seed dispersers access to important resources such as nectar, oils, and pollen for food (Vamosi et al., 2018; Xiao et al., 2021).

### 4.2 Traits of families with high and low species richness

Based on strict consensus criteria, we identified that 75 angiosperm families are species poor, and 12 had high SR (**Supplementary Data File 4**). Poor SR families were less likely to have zygomorphic flowers and more likely to have spiral flowers—a finding that is consistent with previous evidence showing that lineages with zygomorphic flowers have elevated rates of diversification potentially driven by plant–pollinator coevolution (Cubas, 2004; Vamosi and Vamosi, 2010). Families with poor SR were also more likely to be dioecious, an association that has been debated, but is consistent with some studies that have argued that the higher extinction and/or lower speciation rates may lead to lower SR of dioecious clades (Heilbuth, 2000; Kay et al., 2006b), an interesting observation given recent evidence that dioecious taxa are more common at higher latitudes (Wang et al., 2021). It should be noted, however, that we only considered families with exclusively dioecious taxa to be dioecious: the relationship between sexual system and DR deserves further attention, especially at lower taxonomic levels (see Sabath et al., 2016). Additionally, we find that families with poor SR were less likely to exhibit biotic fertilization or seed dispersal (lower rates of vertebrate fertilization and insect dispersal); were less likely to have an annual life history, climbing growth habit, or epiphytic taxa; and had a lower mean seed weight (discussed below). Lastly, and consistent with previous studies (Tank et al., 2015; Landis et al., 2018; Ren et al., 2018), our results suggest that families with poor SR are less likely to have undergone an ancestral WGD event while families with high SR are both more likely to have experienced an ancestral WGD and to harbor polyploid taxa. Despite broad evidence that WGD events are associated with lineages exhibiting high DRs in angiosperms, difficulty in placing the timing of WGD events on a phylogenetic tree and tracking how polyploidization directly influenced angiosperm trait evolution continues to be a matter of debate (Clark and Donoghue, 2018; Vamosi et al., 2018; Leebens-Mack et al., 2019).

In contrast to the large number of families with low levels of SR, we find a relatively small number of angiosperm families that exceeded the albeit high rate of diversification in angiosperm families overall. These families, in addition to being cosmopolitan, have a number of interesting characteristics. Families with high SR harbor a higher proportion of annual taxa, which is consistent with the positive relationship between short generation time and rate of evolution (Aarssen et al., 2006; Crepet and Niklas, 2009). Secondly, families with high SR have a higher proportion of epiphytic taxa, typified by families such as the Orchidaceae and Bromeliaceae; the ability to live in the upper parts of the forest canopy for flowering taxa likely opened new niches with fewer competitors. Third, families with high SR were more likely to have zygomorphic flowers and have SI systems. Families with high SR exhibit large variation in floral morphology and life-history traits (e.g., Fababaeae, Rubiaceae, Asteracee, Poaceae, and Orchidaceae), two factors that have likely been important for their success (Benton et al., 2022). Furthermore, in all of the families with high SR, between 20% and 80% of the taxa for which data were available were identified as self-incompatible or self-sterile and four of these families have SSI, a significantly higher proportion than expected based on the fact that only 17 angiosperm families are exclusively SSI overall (see Ferrer and Good, 2012). Self-incompatible lineages may have historically had high DRs because, in addition to preventing inbreeding, species with SI maintain large effective population sizes due to the negative frequency-dependent selection operating at the S-locus, which serves to maintain connectivity between geographically distant populations and allows for greater opportunities for selection (due to a large N_e_) across broad geographic and temporal time scales. These factors are expected to reduce the extinction rate of self-incompatible lineages, and may be associated with high DRs, an association that deserves further attention.

### 4.3 Biogeographic correlates of angiosperm diversity

In this study, we generated a new database of information regarding the global distribution of flowering plant families and find that families with high SR were exclusively found in 6–8 of the 8 biomes and 11–14 of the 14 realms, while those with poor SR were more likely to be found in a single or a few biomes, and 1–4 of the 14 realms. Thus, biogeographic location appears to be an important factor restricting depauperons. These observations are similar to those made in a study by Vamosi and Vamosi (2011) that found that available area was by far the strongest predictor of SR, explaining ~50% of variation in family SR, while tropical habitat *per se* was not a predictor of SR.

Ramírez-Barahona et al. (2020) examined the stem and crown age of angiosperm families in arid, temperate, and tropical biomes and find that the stem age of angiosperm families in tropical biomes is older than that of families in arid and temperate biomes, a so-called “out of the tropics” scenario of angiosperm diversification. In our investigation of the biomes of 75 families exhibiting poor SR, we find that they were more likely to have localized and highly localized distributions and were predominantly found in the Neotropic, Afrotropic, and Australasia realms. They were also more likely to be found in one or a few biomes, but were only statistically detected to be overrepresented in Tropical and Subtropical Moist Broadleaf Forests biomes. However, visualization of the mean DR of georeferenced taxa belonging to families with poor SR indicates that they are non-randomly distributed in the world.

In a recent study, Hagen et al. (2021) performed a detailed analyses of the factors driving the diversity in tropical moist forests. Tropical moist forests harbor much of the world’s biodiversity, but their diversity is not evenly distributed; they are more diverse in the neotropics and Indomalaya than in the Afrotropics. By simulating paleoenvironmental dynamics and macroevolutionary rates, they show that differences in mountain building, aridification (in Africa), and global temperature fluxes shaped historical rates of speciation and extinction resulting in pantropical diversity disparity in both plants and animals. Collectively, this suggests that some of the depauperons we identified belong to families that are less widely distributed and located in ecozones/realms that have overall lower diversity. However, we also find families with poor SR in the neotropics, even though it is home to all of the most species-rich families.

In conclusion, we find that ~1/3 of all angiosperm families are species poor. Intriguingly, the 75 families with poor SR collectively harbor a mere 196 species of the 207,801 species represented by the 248 families included in this study. The families with poor SR have uniformly very low DRs hovering between −0.04 and +0.04 speciation events per million years and appear to be non-randomly distributed around the globe. On the other hand, families with high SR have mean DRs between 0.11 and 0.25 and dominate in mountainous regions and in grasslands (logical since 2 of the 12 high SR families are grasses), though a full analysis is wanting. Although some studies have shown a lack of relationship between SR and DR (Tietje et al., 2022), the manner in which we approached this problem, by first identifying families with high or low SR and then plotting the average DRs of georeferenced taxa belonging to those families, provides a somewhat unique approach to elucidating the evolutionary dynamics of families with unusually low or high DRs. Nevertheless, to understand the evolutionary persistence of depauperons and the broad factors favoring the diversification of angiosperm lineages, further analyses are required. The availability of new geospatial data on the distribution of angiosperms combined with efforts to sequence the ToL should shed light on the particular challenges and roles of plant lineages in responding to future climate change scenarios.

## Data availability statement

The original contributions presented in the study are included in the article/**Supplementary Material**. Further inquiries can be directed to the corresponding author.

## Author contributions

MF: Conceptualization, Formal analysis, Methodology, Visualization, Writing – original draft, Writing – review & editing, Data curation, Investigation. MV-C: Data curation, Formal analysis, Methodology, Visualization, Writing – review & editing. TH: Data curation, Methodology, Resources, Supervision, Writing – review & editing. SG: Formal analysis, Methodology, Visualization, Writing – review & editing, Conceptualization, Funding acquisition, Project administration, Supervision, Writing – original draft.

## Supplementary material

Supplementary Data Sheet 1 Scripts to run R and maple code (.docx).

Supplementary Data Sheet 2
**Supplementary Tables S1–S17** (.docx).

Supplementary Data Sheet 3
**Supplementary Figures S1–S3** (.pdf).

Supplementary Data Sheet 4 Excel tables describing the date of divergence of angiosperm families inferred from all five studies employed in this analysis, the classification of family species richness (SR), and categorization/value for all traits and geographical distribution examined.
