# Corrigendum: Geographical and life-history traits associated with low and high species richness across angiosperm families

**DOI:** 10.3389/fpls.2024.1378611

**Published:** 2024-02-07

**Authors:** Miriam Monserrat Ferrer, Marilyn Vásquez-Cruz, Tania Hernández-Hernández, Sara V. Good

**Affiliations:** ^1^ Departamento de Manejo y Conservacion de Recursos Naturales Tropicales, Universidad Autonoma de Yucatan, Merida Yucatan, Mexico; ^2^ Tecnologico Nacional de Mexico/ITS Irapuato, Guanajuato, Mexico; ^3^ Research and Collections, Desert Botanical Garden, Phoenix, AZ, United States; ^4^ Department of Biology, The University of Winnipeg, Winnipeg, MB, Canada

**Keywords:** diversification rates, depauperons, self-incompatibility, floral traits, life-history traits, whole genome duplication event, growth habit

In the published article, an author name was incorrectly written as Miriam Montserrat Ferrer. The correct spelling is Miriam Monserrat Ferrer.

The authors apologize for this error and state that this does not change the scientific conclusions of the article in any way. The original article has been updated.

