# Supplementary material for: Geographical and life-history traits associated with low and high species richness across angiosperm families
Source: Front Plant Sci. 2023 Nov 24;14:1276727. doi: 10.3389/fpls.2023.1276727 (PMC10722503; doi:10.3389/fpls.2023.1276727)
Supplement: Supplementary Data Sheet 1 — Scripts to run R and maple code (.docx). [file DataSheet_1.pdf]

Geographical and life-history traits associated with low and high species richness across angiosperm families

Supplementary Data File 1

TESS analysis

The priors were set to: sampling fraction = number of nodes in the tree + 1, no. exp.mass.extinctions = 2, no. expected rate changes = 3, speciation.rate.mean.prior = 0.2, speciation.rate.stdev.prior=0.5, extinction.mean.prior = 0.15; extinction.stdev.prior=0.5, expected survival.probability=0.05; max_iterations=500,000.

```
#load libraries
library(TESS)
library(Rcpp)
library(phytools)
library(ggplot2)
library(ape)

#Example of TESS analysis with the Ramirez data set.

#1_ Trim trees to remove non-angiosperm taxa.
#import phylogeny
ramirez<- read.nexus("RC_complete_MCCv_2.tre")
#Then create list of species to trim
species<-
c("Gnetales_Welwitschiaceae_Welwitschia_spp","Gnetales_Gnetaceae_Gnetum_spp","Cycadales_Cyca
daceae_Cycas_spp","Ginkgoales_Ginkgoaceae_Ginkgo_spp","Pinales_Cupressaceae_Metasequoia_glypt
ostroboides","Pinales_Pinaceae_Pinus_spp")
#Then prune those species.
pruned.ramirez <-drop.tip(ramirez,ramirez$tip.label[match(species, ramirez$tip.label)])
#Then write the new trimmed tree to file
write.tree(pruned.ramirez)
#Then check if the tree is ultrametric
is.ultrametric(pruned.ramirez)
#If it fails test, use be.ultrametric function to force to be ultrametric
be.ultrametric((phy=ramirez))

#Script to test for shifts in DR over time using TESS
# SETTING THE LIMITS FOR THE TESS CALCULATION
samplingFraction <- (bell_e$Nnode + 1) /322
numExpectedMassExtinctions <- 2
numExpectedRateChanges <- 2
speciationPriorMu <- 0.2
speciationPriorSigma <- 0.5
extinctionPriorMu <- 0.15
extinctionPriorSigma <- 0.5
speciationRatePriorMean <- log((speciationPriorMu^2)
                    /sqrt(speciationPriorSigma^2+
```

```
                                speciationPriorMu^2))
speciationRatePriorStDev <- sqrt( log(1+speciationPriorSigma^2
                               /(speciationPriorMu^2)))
# Transform the priors on the extinction rate into log space.
extinctionRatePriorMean <- log((extinctionPriorMu^2)
                           /sqrt(extinctionPriorSigma^2+
                                extinctionPriorMu^2))
extinctionRatePriorStDev <- sqrt( log(1+extinctionPriorSigma^2
                               /(extinctionPriorMu^2)))
expectedSurvivalProbability <- 0.05

pMassExtinctionPriorShape2 <- 100
pMassExtinctionPriorShape1 <- - pMassExtinctionPriorShape2 *
  expectedSurvivalProbability /
  (expectedSurvivalProbability - 1)


curve(dbeta(x,shape1=pMassExtinctionPriorShape1,
          shape2=pMassExtinctionPriorShape2),n=1001,
     xlab='survival probability',ylab='density',las=1)
# Plot the 95% prior interval on the survival probability.
abline(v = qbeta(c(0.025,0.975),shape1=pMassExtinctionPriorShape1,
              shape2=pMassExtinctionPriorShape2),lty=2)
getwd()
set.seed(14235)
tess.analysis(ramirez,
           empiricalHyperPriors = FALSE,
           initialSpeciationRate = speciationPriorMu,
           speciationRatePriorMean = speciationRatePriorMean,
           speciationRatePriorStDev = speciationRatePriorStDev,
           initialExtinctionRate = extinctionPriorMu,
           extinctionRatePriorMean = extinctionRatePriorMean,
           extinctionRatePriorStDev = extinctionRatePriorStDev,
           samplingProbability = samplingFraction,
           numExpectedRateChanges = numExpectedRateChanges,
           numExpectedMassExtinctions = numExpectedMassExtinctions,
           pMassExtinctionPriorShape1 = pMassExtinctionPriorShape1,
           pMassExtinctionPriorShape2 = pMassExtinctionPriorShape2,
           MAX_ITERATIONS = 500000,
           dir = "tess_analysis_bell")
output <- tess.process.output("tess_analysis_ramirez",
                       numExpectedRateChanges = numExpectedRateChanges,
                       numExpectedMassExtinctions = numExpectedMassExtinctions)


layout.mat <- matrix(1:4,nrow=1,ncol=4,byrow=TRUE)
par(mar=c(3, 3, 3, 3))
layout(layout.mat)
```

```
tess.plot.output(output,
             fig.types = c("speciation rates",
                          "speciation shift times",
                          "extinction rates",
                          "extinction shift times"),
    #                      "mass extinction Bayes factors",
    #                      "mass extinction times"),
             las=2)
```

****

BubblePlots

```
##loading libraries

library(ggplot2)
library(reshape2)

##importing data for all biomes

bp = read.csv("biome.csv", header = TRUE)
bpm = melt(bp, id = c("biome"))

##setting colors and factors for all biomes
colours = c( "red",  "darkgreen", "blue", "black")
bpm$biome <- factor(bpm$biome,levels=unique(bpm$biome))

##bubble plot for all biomes
bpx = ggplot(bpm, aes(x = biome, y = variable)) +
  geom_point(aes(size = value, fill = variable), alpha = 0.75, shape = 21) +
  scale_size_continuous(limits = c(0.000001, 100), range = c(1,18), breaks = c(1,10,50,75,100)) +
  labs( x= "", y = "", title= "Biome", size = "Relative Abundance (%)", fill = "")  +
  theme(legend.key=element_blank(),
  axis.text.x = element_text(colour = "black", size = 12, face = "bold", angle = 90, vjust = 0.3, hjust = 1),
  axis.text.y = element_text(colour = "black", face = "bold", size = 11),
  legend.text = element_text(size = 10, face ="bold", colour ="black"),
  legend.title = element_text(size = 12, face = "bold"),
  panel.background = element_blank(), panel.border = element_rect(colour = "black", fill = NA, size =
1.2),
  legend.position = "right") +
  scale_fill_manual(values = colours, guide = FALSE) +
  scale_y_discrete(limits = rev(levels(bpm$variable)))

bpx
###Exported with Rstudio
```

```
##importing data for perianth symmetry

fs = read.csv("florals.csv", header = TRUE)
fsm = melt(fs, id = c("florals"))

##setting colors and factors for perianth symmetry
colours = c( "red",  "darkgreen", "blue", "black")
fsm$florals <- factor(fsm$florals,levels=unique(fsm$florals))

##bubble plot for perianth symmetry
fsx = ggplot(fsm, aes(x = florals, y = variable)) +
  geom_point(aes(size = value, fill = variable), alpha = 0.75, shape = 21) +
  scale_size_continuous(limits = c(0.000001, 100), range = c(1,12), breaks = c(1,10,50,75,100)) +
  labs( x= "", y = "", title= "Perianth symmetry", size = "Relative Abundance (%)", fill = "")  +
  theme(legend.key=element_blank(),
  axis.text.x = element_text(colour = "black", size = 12, face = "bold", angle = 90, vjust = 0.3, hjust = 1),
  axis.text.y = element_text(colour = "black", face = "bold", size = 11),
  legend.text = element_text(size = 10, face ="bold", colour ="black"),
  legend.title = element_text(size = 12, face = "bold"),
  panel.background = element_blank(), panel.border = element_rect(colour = "black", fill = NA, size =
1.2),
  legend.position = "right") +
  scale_fill_manual(values = colours, guide = none) +
  scale_y_discrete(limits = rev(levels(fsm$variable)))

fsx

dr = read.csv("distribrange.csv", header = TRUE)
drm = melt(dr, id = c("distribrange"))

colours = c( "red",  "darkgreen", "blue", "black")

drm$distribrange <- factor(drm$distribrange,levels=unique(drm$distribrange))

drx = ggplot(drm, aes(x = distribrange, y = variable)) +
  geom_point(aes(size = value, fill = variable), alpha = 0.75, shape = 21) +
  scale_size_continuous(limits = c(0.000001, 100), range = c(1,20), breaks = c(1,10,50,75)) +
  labs( x= "", y = "", title= "Distribution range", size = "Relative Abundance (%)", fill = "")  +
  theme(legend.key=element_blank(),
  axis.text.x = element_text(colour = "black", size = 12, face = "bold", angle = 90, vjust = 0.3, hjust = 1),
  axis.text.y = element_text(colour = "black", face = "bold", size = 11),
  legend.text = element_text(size = 10, face ="bold", colour ="black"),
  legend.title = element_text(size = 12, face = "bold"),
  panel.background = element_blank(), panel.border = element_rect(colour = "black", fill = NA, size =
1.2),
  legend.position = "right") +
  scale_fill_manual(values = colours, guide = none) +
  scale_y_discrete(limits = rev(levels(drm$variable)))
```

```
##importing data for all realms

rp = read.csv("realm.csv", header = TRUE)
rpm = melt(rp, id = c("realm"))

##setting colors and factors for all realms
colours = c( "red",  "darkgreen", "blue", "black")
rpm$realm <- factor(rpm$realm,levels=unique(rpm$realm))

##bubble plot for all biomes
rpx = ggplot(rpm, aes(x = realm, y = variable)) +
  geom_point(aes(size = value, fill = variable), alpha = 0.75, shape = 21) +
  scale_size_continuous(limits = c(0.000001, 100), range = c(1,24), breaks = c(1,10,50,75,100)) +
  labs( x= "", y = "", title= "Realms", size = "Relative Abundance (%)", fill = "")  +
  theme(legend.key=element_blank(),
  axis.text.x = element_text(colour = "black", size = 12, face = "bold", angle = 90, vjust = 0.3, hjust = 1),
  axis.text.y = element_text(colour = "black", face = "bold", size = 11),
  legend.text = element_text(size = 10, face ="bold", colour ="black"),
  legend.title = element_text(size = 12, face = "bold"),
  panel.background = element_blank(), panel.border = element_rect(colour = "black", fill = NA, size =
1.2),
  legend.position = "right") +
  scale_fill_manual(values = colours, guide = 'none') +
  scale_y_discrete(limits = rev(levels(rpm$variable)))

rpx
```

Maple script

```
array of taxon ages: times
:=[t1,t2,t3,….tn]:
corresponding array of species numbers: species
:=[s1,s2,s3,…sn]:
> for lambda from 0.1 by 0.01 to 100 do for r from 0.0001 by 0.0002 to 0.2 do
> pSp := 0;
> for i from 1 to n do t := times[i]; s := species[i]; mu := lambda -r; beta :=
> lambda*(exp((lambda-mu)*t)-1) /(lambda*exp((lambda-mu)*t)-mu); pSpi :=
> evalf(ln(1-beta)+(s-1)*ln(beta)); pSp := pSp+pSpi;
> end do; print (lambda,mu,pSp); end do; end do;
```
