# Supplementary material for: Geographical and life-history traits associated with low and high species richness across angiosperm families
Source: Front Plant Sci. 2023 Nov 24;14:1276727. doi: 10.3389/fpls.2023.1276727 (PMC10722503; doi:10.3389/fpls.2023.1276727)
Supplement: Supplementary Data Sheet 2 — Supplementary Tables S1–S17 (.docx). [file DataSheet_2.pdf]

Supplementary Data File 2

Tables 1-10

Miriam M. Ferrer, Marylin Vázquez-Cruz,Tania Hernández-Hernández, Sara V. Good.

Geographical and life-history traits associated with low and high species richness across angiosperm families

Table S1. Temporal periods according to the number of shifts + 1 according the median age for a shift in the speciation or extinction rate based on the five calibrated phylogenies: Wikström et al. (2001), Bell et al. (2010), Hernández-Hernández and Wiens (2020), Li et al. (2019), and Ramírez-Barahona et al. (2020).

**Wikstrom et al. (2001)**	**Bell et al. (2010)**	**Hernández-Hernández and Wiens (2020)**	**Li et al. (2019)**	**Ramírez-Barahona et al. (2020)**
0-43	0-23	0-14.4	0-24.49	0-21
44-69	24-38	14.5-60	24.49-45	21.1-40
70-92	39-59	60.1-86	45.1-60	40.1-57.7
93-120	60-77	86.1-104.2	60-200	57.8-80
121-132	78-98	104.3-117.8		80.1-103
	99-128	117.9-130		103.1-195.8
	128-136			

Table S2. Estimates for the parameters of a birth-death diversification model: λ, μ, (speciation and extinction rate), pSp (likelihood of the model), r (diversification rate) and ε (extinction fraction λ/μ) obtained using a maximum likelihood approach (Bokma, 2003) for 432 angiosperm families (All) and those that were originated during different periods in which shifts on the diversification rates occurred based on their crown ages from five calibrated phylogenies: Wikström et al. (2001), Bell et al. (2010), Hernández-Hernández and Wiens (2020), Li et al. (2019), and Ramírez-Barahona et al. (2020) and the number of species from the APG website (Stevens, 2021).

**Period (MYBP)**	**λ**	**μ**	**pSp**	**r**	**ε**	**Estimate**
Wikström et al. (2001)						
**0-43**	25.01	25.01	-655.85	0.0001	1.0000	MLE
**0-43**	18.63	18.62	-656.85	0.0141	0.9992	max
**0-43**	29.17	29.17	-656.85	0.0001	1.0000	min
**44-69**	14.69	14.69	-1486.40	0.0001	1.0000	MLE
**44-69**	13.11	13.11	-1487.40	0.0009	0.9999	max
**44-69**	16.31	16.31	-1487.39	0.0001	1.0000	min
**70-92**	6.14	6.14	-785.83	0.0001	1.0000	MLE
**70-92**	5.14	5.14	-786.82	0.0029	0.9994	max
**70-92**	7.04	7.04	-786.81	0.0001	1.0000	min
**93-120**	0.68	0.66	-237.49	0.0199	0.9707	MLE
**92-120**	0.55	0.53	-238.48	0.0197	0.9642	max
**92-120**	1.15	1.14	-238.48	0.0131	0.9886	min
**121-132**	0.76	0.76	-56.18	0.0001	0.9999	MLE
**121-132**	0.48	0.48	-57.14	0.0009	0.9981	max
**121-132**	1.23	1.23	-57.17	0.0001	0.9999	min
**All**	13.16	13.1599	-3324.593	0.0001	1.0000	MLE
**All**	12.32	12.3199	-3325.592	0.0001	1.0000	max
**All**	14.11	14.1099	-3325.59	0.0001	1.0000	min
Bell et al. (2010)						
**0-23**	42.75	42.75	-323.60	0.0001	1.0000	MLE
**0-23**	30.75	30.73	-324.60	0.0186	0.9994	max
**0-23**	53.50	53.50	-324.60	0.0001	1.0000	min
**24-38**	25.20	25.20	-600.21	0.0001	1.0000	MLE
**24-38**	18.15	18.13	-601.21	0.0157	0.9991	max
**24-38**	29.75	29.75	-601.20	0.0001	1.0000	min
**39-59**	23.13	23.13	-1058.84	0.0001	1.0000	MLE
**39-59**	20.22	20.22	-1059.84	0.0023	0.9999	max
**39-59**	26.15	26.15	-1059.82	0.0001	1.0000	min
**60-77**	7.35	7.35	-690.70	0.0001	1.0000	MLE

**60-77**	5.40	5.39	-691.70	0.0079	0.9985	max
**60-77**	8.51	8.51	-691.69	0.0001	1.0000	min
**78-98**	2.32	2.32	-474.21	0.0001	1.0000	MLE
**78-98**	1.78	1.78	-475.20	0.0047	0.9974	max
**78-98**	2.74	2.74	-475.18	0.0001	1.0000	min
**99-128**	0.06	0.05	-18.36	0.0109	0.8183	MLE
**99-128**	0.04	0.03	-19.31	0.0091	0.7725	max
**99-128**	0.24	0.24	-19.35	0.0003	0.9988	min
**128-136**	0.02	0.01	-4.47	0.0057	0.6200	MLE
**128-136**	0.01	0.00	-5.46	0.0059	-0.1800	max
**128-136**	0.08	0.08	-5.47	0.0001	0.9988	min
**All**	17.85	17.85	-3364.04	0.0001	1.0000	MLE
**All**	16.70	16.70	-3365.03	0.0001	1.0000	max
**All**	19.13	19.13	-3365.03	0.0001	1.0000	min
Hernández-Hernández and Wiens (2020)						
**0-14.4**	132.60	132.60	-24.03	0.0001	1.0000	MLE
**0-14.4**	29.90	29.60	-25.03	0.2960	0.9901	max
**0-14.4**	343.20	343.20	-25.03	0.0001	1.0000	min
**14.5-60**	16.15	16.15	-1847.35	0.0001	1.0000	MLE
**14.5-60**	14.57	14.57	-1848.34	0.0010	0.9999	max
**14.5-60**	17.66	17.66	-1848.34	0.0001	1.0000	min
**60.1-86**	6.98	6.98	-940.39	0.0047	0.9993	MLE
**60.1-86**	5.28	5.27	-941.39	0.0120	0.9977	max
**60.1-86**	6.98	6.97	-941.29	0.0079	0.9989	min
**86.1-104.2**	3.41	3.39	-245.52	0.0250	0.9927	MLE
**86.1-104.2**	0.50	0.45	-246.52	0.0516	0.8968	max
**86.1-104.2**	4.46	4.44	-246.52	0.0250	0.9944	min
**104.3-117.8**	4.34	4.33	-112.72	0.0060	0.9986	MLE
**104.3-117.8**	3.08	3.07	-113.71	0.0060	0.9981	max
**104.3-117.8**	8.48	8.48	-113.71	0.0010	0.9999	min
**117.9-130**	0.28	0.28	-45.59	0.0001	0.9996	MLE
**117.9-130**	0.18	0.18	-46.55	0.0007	0.9961	max
**117.9-130**	0.46	0.46	-46.59	0.0001	0.9998	min
**All**	13.75	13.75	-3290.04	0.0001	1.0000	MLE
**All**	12.86	12.86	-3291.04	0.0001	1.0000	max
**All**	14.73	14.73	-3291.04	0.0001	1.0000	min
Li et al. (2019)						
**0-24.49**	0.21	0.14	-4.09	0.0680	0.6762	MLE
**0-24.49**	0.07	0.01	-5.08	0.0600	0.1429	max
**0-24.49**	1.13	1.13	-5.09	0.0019	0.9983	min

**24.49-45**	13.44	13.44	-554.35	0.0001	1.0000	MLE
**24.49-45**	9.10	9.08	-555.35	0.0210	0.9977	max
**24.49-45**	15.82	15.82	-555.34	0.0001	1.0000	min
**45.1-60**	9.46	9.46	-469.78	0.0010	0.9999	MLE
**45.1-60**	2.42	2.38	-470.78	0.0450	0.9814	max
**45.1-60**	11.32	11.32	-470.77	0.0010	0.9999	min
**60-200**	9.32	9.32	-2205.28	0.0001	1.0000	MLE
**60-200**	8.54	8.54	-2206.27	0.0007	0.9999	max
**60-200**	10.15	10.15	-2206.27	0.0001	1.0000	min
**All**	10.08	10.0799	-3242.63	0.0001	1.0000	MLE
**All**	9.44	9.4399	-3243.576	0.0001	1.0000	MAX
**All**	10.78	10.7799	-3243.589	0.0001	1.0000	MIN
Ramírez-Barahona et al. (2020)						
**0-21**	1.86	1.83	-324.89	0.0299	0.9839	MLE
**0-21**	1.59	1.56	-325.89	0.0293	0.9816	max
**0-21**	2.50	2.50	-325.89	0.0001	1.0000	min
**21.1-40**	4.14	4.14	-600.92	0.0001	1.0000	MLE
**21.1-40**	2.62	2.59	-601.91	0.0270	0.9897	max
**21.1-40**	4.77	4.77	-601.90	0.0001	1.0000	min
**40.1-57.7**	9.27	9.27	-699.28	0.0001	1.0000	MLE
**40.1-57.7**	5.36	5.34	-700.27	0.0190	0.9965	max
**40.1-57.7**	10.73	10.73	-700.27	0.0001	1.0000	min
**57.8-80**	19.15	19.15	-955.46	0.0001	1.0000	MLE
**57.8-80**	13.31	13.30	-956.46	0.0090	0.9993	max
**57.8-80**	21.86	21.86	-956.46	0.0001	1.0000	min
**80.1-103**	0.09	0.00	-196.85	0.0860	0.0444	MLE
**80.1-103**	0.09	0.00	-197.40	0.0840	0.0118	max
**80.1-103**	4.05	4.02	-197.84	0.0330	0.9919	min
**103.1-195.8**	18.91	18.91	-132.10	0.0001	1.0000	MLE
**103.1-195.8**	12.47	12.47	-133.10	0.0050	0.9996	max
**103.1-195.8**	27.91	27.91	-133.10	0.0001	1.0000	min
**All**	2.73	2.69	-2961.33	0.0400	0.9853	MLE
**All**	2.48	2.44	-2963.32	0.0400	0.9839	max
**All**	3.00	2.96	-2963.24	0.0400	0.9867	min

MLE: maximum likelihood estimate, max: maximum value of r, min: minimum value of r

Table S3. Estimates for the parameters of a birth-death diversification model: λ, μ, (speciation and extinction rate), pSp (likelihood of the model), r (diversification rate) and ε (extinction fraction λ/μ) obtained using a maximum likelihood approach (Bokma, 2003) for all angiosperm families and those that were originated during different periods in which shifts on the diversification rates occurred based on their crown ages from five calibrated phylogenies: Wikström et al. (2001), Bell et al. (2010), Hernández-Hernández and Wiens (2020), Li et al. (2019), and Ramírez-Barahona et al. (2020) and the number of species from the APG website (Stevens, 2021).

Period (MYBP)	λ	μ	pSp	r	ε	Estimate
Wikström et al. 2001						
**0-43**	36.90	36.90	-455.39	0.0001	1.0000	MLE
**0-43**	27.00	26.99	-456.39	0.0145	0.9995	max
**0-43**	45.08	45.08	-456.39	0.0001	1.0000	min
**44-69**	19.56	19.56	-1098.34	0.0001	1.0000	MLE
**44-69**	16.66	16.66	-1099.34	0.0019	0.9999	max
**44-69**	22.10	22.10	-1099.33	0.0001	1.0000	min
**70-92**	9.40	9.40	-496.53	0.0001	1.0000	MLE
**70-92**	7.44	7.44	-497.52	0.0029	0.9996	max
**70-92**	11.24	11.24	-497.51	0.0001	1.0000	min
**93-120**	0.50	0.47	-186.02	0.0280	0.9440	MLE
**92-120**	0.50	0.48	-186.85	0.0250	0.9500	max
**92-120**	0.97	0.95	-187.02	0.0190	0.9804	min
**121-132**	0.76	0.76	-56.18	0.0001	0.9999	MLE
**121-132**	0.48	0.48	-57.14	0.0009	0.9981	max
**121-132**	1.23	1.23	-57.17	0.0001	0.9999	min
**All**	18.42	18.42	-2374.21	0.0001	1.0000	MLE
**All**	17.00	17.00	-2375.21	0.0001	1.0000	max
**All**	20.04	20.04	-2375.21	0.0001	1.0000	min
Bell et al. 2010						
**0-23**	34.90	34.88	-277.05	0.0201	0.9994	MLE
**0-23**	33.76	33.74	-277.05	0.0246	0.9993	max
**0-23**	65.20	65.20	-277.04	0.0001	1.0000	min
**24-38**	30.35	30.35	-431.94	0.0001	1.0000	MLE
**24-38**	21.30	21.28	-432.94	0.0156	0.9993	max
**24-38**	36.90	36.90	-432.94	0.0001	1.0000	min
**39-59**	32.95	32.95	-735.73	0.0001	1.0000	MLE
**39-59**	26.85	26.84	-736.72	0.0060	0.9998	max
**39-59**	38.50	38.50	-736.72	0.0001	1.0000	min
**60-77**	4.20	4.20	-256.03	0.0001	1.0000	MLE

**60-77**	2.85	2.84	-256.97	0.0071	0.9975	max
**60-77**	5.34	5.34	-257.02	0.0001	1.0000	min
**78-98**	8.65	8.65	-449.16	0.0001	1.0000	MLE
**78-98**	5.45	5.44	-450.11	0.0091	0.9983	max
**78-98**	10.45	10.45	-450.16	0.0001	1.0000	min
**99-128**	0.03	0.01	-18.13	0.0209	0.3033	MLE
**99-128**	0.02	0.00	-19.10	0.0183	0.0850	max
**99-128**	0.21	0.21	-19.11	0.0007	0.9967	min
**128-136**	0.06	0.05	-18.36	0.0109	0.8183	MLE
**128-136**	0.04	0.03	-19.31	0.0091	0.7725	max
**128-136**	0.24	0.24	-19.35	0.0003	0.9988	min
**All**	25.64	25.64	-2358.64	0.0001	1.0000	MLE
**All**	23.64	23.64	-2359.63	0.0001	1.0000	max
**All**	27.88	27.88	-2359.63	0.0001	1.0000	min
Hernández-Hernández and Wiens (2020)						
**0-14.4**	132.60	132.60	-24.03	0.0001	1.0000	MLE
**0-14.4**	29.90	29.60	-25.03	0.2960	0.9901	max
**0-14.4**	343.20	343.20	-25.03	0.0001	1.0000	min
**14.5-60**	16.88	16.88	-1763.70	0.0001	1.0000	MLE
**14.5-60**	15.10	15.10	-1764.69	0.0021	0.9999	max
**14.5-60**	18.58	18.58	-1764.68	0.0001	1.0000	min
**60.1-86**	9.54	9.54	-824.76	0.0001	1.0000	MLE
**60.1-86**	6.24	6.23	-825.75	0.0111	0.9982	max
**60.1-86**	10.96	10.96	-825.76	0.0001	1.0000	min
**86.1-104.2**	3.41	3.39	-245.52	0.0250	0.9927	MLE
**86.1-104.2**	0.50	0.45	-246.52	0.0516	0.8968	max
**86.1-104.2**	4.46	4.44	-246.52	0.0250	0.9944	min
**104.3-117.8**	4.34	4.33	-112.72	0.0060	0.9986	MLE
**104.3-117.8**	3.08	3.07	-113.71	0.0060	0.9981	max
**104.3-117.8**	8.48	8.48	-113.71	0.0010	0.9999	min
**117.9-130**	0.28	0.28	-45.59	0.0001	0.9996	MLE
**117.9-130**	0.18	0.18	-46.55	0.0007	0.9961	max
**117.9-130**	0.46	0.46	-46.59	0.0001	0.9998	min
**All**	14.70	14.70	-3072.72	0.0001	1.0000	MLE
**All**	13.72	13.72	-3073.68	0.0001	1.0000	max
**All**	15.76	15.76	-3073.68	0.0001	1.0000	min
LI et al. 2019						
**0-24.49**	0.21	0.14	-4.09	0.0680	0.6762	MLE
**0-24.49**	0.07	0.01	-5.08	0.0600	0.1429	max
**0-24.49**	1.13	1.13	-5.09	0.0019	0.9983	min

**24.49-45**	15.44	15.44	-494.13	0.0001	1.0000	MLE
**24.49-45**	12.00	11.99	-495.01	0.0090	0.9993	max
**24.49-45**	18.36	18.36	-495.13	0.0001	1.0000	min
**45.1-60**	10.64	10.64	-431.73	0.0001	1.0000	MLE
**45.1-60**	2.12	2.07	-432.69	0.0490	0.9769	max
**45.1-60**	12.86	12.86	-432.73	0.0001	1.0000	min
**60-200**	11.82	11.82	-1766.56	0.0001	1.0000	MLE
**60-200**	10.68	10.68	-1767.55	0.0007	0.9999	max
**60-200**	12.98	12.98	-1767.53	0.0001	1.0000	min
**All**	12.26	12.26	-2704.21	0.0001	1.0000	MLE
**All**	11.36	11.36	-2705.20	0.0001	1.0000	MAX
**All**	13.20	13.20	-2705.20	0.0001	1.0000	MIN
Ramírez-Barahona et al. 2020						
**0-21**	2.08	2.05	-269.07	0.0291	0.9860	MLE
**0-21**	1.74	1.71	-270.01	0.0291	0.9833	max
**0-21**	2.60	2.59	-270.07	0.0131	0.9950	min
**21.1-40**	4.88	4.88	-521.83	0.0001	1.0000	MLE
**21.1-40**	3.72	3.71	-522.80	0.0091	0.9976	max
**21.1-40**	5.70	5.70	-522.81	0.0001	1.0000	min
**40.1-57.7**	9.98	9.98	-655.34	0.0001	1.0000	MLE
**40.1-57.7**	8.50	8.50	-656.34	0.0007	0.9999	max
**40.1-57.7**	10.73	10.73	-700.27	0.0001	1.0000	min
**57.8-80**	22.22	22.22	-831.60	0.0001	1.0000	MLE
**57.8-80**	18.60	18.60	-832.59	0.0015	0.9999	max
**57.8-80**	25.65	25.65	-832.60	0.0001	1.0000	min
**80.1-103**	0.97	0.91	-167.34	0.0560	0.9423	MLE
**80.1-103**	0.09	0.00	-167.83	0.0860	0.0444	max
**80.1-103**	14.99	14.97	-167.70	0.0151	0.9990	min
**103.1-195.8**	21.70	21.70	-116.53	0.0001	1.0000	MLE
**103.1-195.8**	14.42	14.42	-117.53	0.0017	0.9999	max
**103.1-195.8**	33.08	33.08	-117.53	0.0001	1.0000	min
**All**	5.12	5.09	-2606.94	0.0251	0.9951	MLE
**All**	4.78	4.75	-2607.83	0.0251	0.9947	max
**All**	5.68	5.65	-2608.88	0.0251	0.9956	min

MLE: maximum likelihood estimate, max: maximum value of r, min: minimum value of r

Table S4. Frequency of families with poor, predicted, high or mixed species richness based on strict consensus across five datasets and tabulated by the number of species in the family. Results are presented for 432 angiosperm families by estimating the crown age of families using the age of their sister family (upper panel) or for the 235 angiosperm families that were included in all five calibrated phylogenies included in our study (lower panel).

**Species richness**	**1**	**2-10**	**11-100**	**101-500**	**501-1000**	**1001-5000**	**5001-26000**	**Total**
	Families with crown ages inferred from sister clade strict consensus							
**unclear**			4					4
**poor**	32	42	1					75
**predicted**		64	117	75	36	49		341
**high**						3	9	12
**Total**	32	106	122	75	36	52	9	432
	Families with crown ages in common across all studies							
**poor**	3	15	1					19
**predicted**		20	56	53	31	46		206
**high**						1	9	10
**Total**	3	35	57	53	31	47	9	235

Table S5. Frequencies of the 432 angiosperm families that were originated during different periods in which shifts on the diversification rates occurred based on their crown ages from five datasets: Wikström et al. (2001), Bell et al. (2010), Hernández-Hernández and Wiens (2020), Li et al. (2019), and Ramírez-Barahona et al. (2020), grouped by the different periods in which shifts on the diversification rates occur. Statistics for a χ^2 independence test are presented, and periods in which more families were found for any of the three classes are presented in red, and those in which less families were found are in blue according to a Haberman residual analysis.

Period (MYBP)	high	low	predicted	Total
Wikström (χ^2 = 6.74, P = 0.5647)				
0-43	7	33	45	85
44-69	11	79	102	192
70-92	8	47	54	109
92-120	3	9	24	36
120-158	1	6	3	10
Total	30	174	228	432
Bell (χ^2 = 62.97, P < 0.0001)				
0-23	4	12	19	35
24-38	6	17	46	69
39-59	8	59	72	139
59-77	2	34	47	83
78-98	4	26	38	68
99-128	14	1	20	35
129-136	0	1	2	3
Total	38	150	244	432
Hernández-Hernández (χ^2 = 5.11, P = 0.7788)				
0-14.4	0	1	2	3
14.5-60	17	98	132	247
60.1-86	9	46	72	127
86.1-104.2	1	15	14	30
104.3-117.8	2	5	8	15
117.9-130	1	2	7	10
Total	30	167	235	432
Li (χ^2 = 4.87, P = 0.7712)				
0-22.4	0	0	2	2
22-5-45-9	6	31	41	78
46-59.9	6	21	38	65
60-200	14	116	157	287
Total	26	168	238	432

Ramírez-Barahona (χ^2 = 18.71, P = 0.0430)				
0-21	8	15	53	76
21.1-40	5	26	72	103
40.1-57.7	6	23	69	98
57.8-80	7	29	81	117
80.1-103	2	8	13	23
103.1-196	1	10	4	15
Total	29	111	292	432

Table S6. Frequencies of the angiosperm families that were originated during different periods in which shifts on the diversification rates occurred based on their crown ages from five calibrated phylogenies: Wikström et al. (2001), Bell et al. (2010), Hernández-Hernández and Wiens (2020), Li et al. (2019), and Ramírez-Barahona et al. (2020), grouped by the different periods in which shifts on the diversification rates occur. Statistics for a χ^2 independence test are presented, and periods in which more families were found for any of the three classes are presented in red, and those in which less families were found are in blue according to a Haberman residual analysis.

Period (MYBP)	high	low	predicted	Total
Wikström et al. 2001 (χ^2 = 11.1474, P = 0.1941)				
**0-43**	5	11	40	56
**44-69**	9	46	82	137
**70-92**	5	25	35	65
**93-120**	2	6	20	28
**121-158**	1	6	3	10
**Total**	22	94	180	286
Bell et al. 2010 (χ^2 = 32.46, P < 0.0012)				
**0-23**	4	6	18	28
**24-38**	6	12	41	59
**39-59**	4	29	60	93
**60-77**	9	12	38	59
**78-98**	2	15	19	36
**99-128**	7	1	8	16
**129-136**	0	1	1	2
**Total**	32	76	185	293
Hernández-Hernández and Wiens 2020 (χ^2 = 4.59, P = 0.9292)				
**0-14.4**	0	1	2	3
**14.5-60**	17	92	125	234
**60.1-86**	8	43	59	110
**86.1-104.2**	1	15	14	30
**104.3-117.8**	2	5	8	15
**117.9-130**	1	2	7	10
**Total**	29	158	215	402
Li et al. 2019 (χ^2 = 3.01, P = 0.8080)				
**0-22.4**	0	0	2	2

**22-5-45-9**	6	24	38	68
**46-59.9**	5	17	37	59
**60-200**	13	75	135	223
**Total**	24	116	212	352
Ramírez-Barahona et al. 2020 (χ^2 = 18.71, P = 0.0430)				
**0-21**	6	7	48	61
**21.1-40**	5	15	67	87
**40.1-57.7**	6	17	67	90
**57.8-80**	6	16	78	100
**80.1-103**	2	6	11	19
**103.1-196**	1	9	3	13
**Total**	26	70	274	370

Table S7. Frequency of 248 families with poor, predicted, or high species richness based on strict consensus tabulated by the absence or presence of fleshy fruits (Vamosi and Vamosi, 2011). Statistics for a χ^2 independence test are presented, and categories in which more families were found for any of the three classes are presented in red, and those in which less families were found are in blue according to a Haberman residual analysis.

**Fleshy fruit**	**poor**	**predicted**	**high**	**Total**
(χ^2 = 0.08082, P = 0.6676)				
**absent**	28	54	3	85
**present**	47	107	9	163
**Total**	75	161	12	248

Table S8. Frequency of 248 families with poor, predicted, or high species richness based on strict consensus tabulated by the absence or presence of different perianth symmetries (Reyes et al., 2016). Statistics for a χ^2 independence test are presented, and categories in which more families were found for any of the three classes are presented in red, and those in which less families were found are in blue according to a Haberman residual analysis.

Floral symmetry	poor	predicted	high	Total
(χ^2 = 35.91, P < 0.0047)				
**Zygomorphic**	6	57	9	72
**Polysymmetric**	56	128	9	193
**Actinomorphic**	2	6	1	9
**Spiral**	7	3	0	10
**Disymmetric**	2	1	1	4
**Asymmetric**		1		1
**Perianthless**	2	4		6
**Monosym metric by reduction**	3	1	1	5
**Total**	78	201	21	300

Table S9. Frequency of self-incompatibility system in 248 families with poor, predicted, or high species richness based on strict consensus tabulated by self-incompability type. Statistics for a χ^2 independence test are presented, and categories in which more families were found for any of the three classes are presented in red, and those in which fewer families were found are in blue according to a Haberman residual analysis.

Type of SI	poor	predicted	high	Total general
(χ^2 = 14.23, P = 0.0499)				
**Stigmatic**	2	7	4	13
**Stylar**	0	12	3	15
**Late acting**	0	14	1	15
**Polymorphic**	0	8	1	9
**SS_unclass**	5	39	0	44
**Homomorphic+Het**	0	12	3	15
**SS_unclass+Het**	0	4	0	4
**Self-compatibility**	8	26	0	34
**Total**	15	122	12	149

Stigmatic: self-incompatibility reaction occurs in stigma, Stylar: self-incompatibility reaction occurs in style, Late acting: self-incompatibility reaction occurs after pollen tube enters the ovary, POL: self-incompatibility reaction occurs in stigma, SS_unclass: self-sterility unclassified; Heterostyly present in the famili.

Table S10. Frequency of 248 families with poor, predicted, or high species richness based on strict consensus tabulated by distribution range. Statistics for a χ^2 independence test are presented, and categories in which more families were found for any of the three classes are presented in red, and those in which fewer families were found are in blue according to a Haberman residual analysis.

Distribution range	poor	predicted	high	Total
(χ^2 = 238.67, P < 0.0001)				
**Highly localized**	20	1	0	21
**Localized**	47	38	0	85
**Widespread**	8	119	3	130
**Cosmopolitan**	0	3	9	12
**Total**	75	161	12	248

Table S11. Frequency of 248 families with poor, predicted, or high species richness based on strict consensus tabulated by distribution pattern. Statistics for a χ^2 independence test are presented, and categories in which more families were found for any of the three classes are presented in red, and those in which less families were found are in blue according to a Haberman residual analysis.

Distibution pattern	poor	predicted	high	Total
(χ^2 = 6.47, P = 0.0393)				
**Continuous**	48	104	12	164
**Disjunct**	27	57	0	84
**Total**	75	161	12	248

Table S12. Frequency of 248 families with poor, predicted, or high species richness based on strict consensus tabulated by realm number. Statistics for a χ^2 independence test are presented, and categories in which more families were found for any of the three classes are presented in red, and those in which less families were found are in blue according to a Haberman residual analysis.

Realm number	poor	predicted	high	Total
(χ^2 = 234.59, P < 0.0001)				
**1**	39	7	0	46
**2 to 4**	32	41	0	73
**5 to 7**	4	113	6	123
8	0	0	6	6
Total	75	161	12	248

Table S13. Frequency of 248 families with poor, predicted, or high species richness based on strict consensus tabulated by realm presence. Statistics for a χ^2 independence test are presented, and categories in which more families were found for any of the three classes are presented in red, and those in which less families were found are in blue according to a Haberman residual analysis.

Realm	poor	predicted	high	Total
(χ^2 = 81.68, P < 0.0001)				
**Afrotropic**	24	127	12	163
**Antartic**	0	0	6	6
**Australasia**	25	121	12	158
**Indomalayan**	23	136	12	171
**Nearctic**	12	121	12	145
**Neotropic**	32	142	12	186
**Oceania**	4	81	12	97
**Palearctic**	20	117	12	149
**Total**	140	845	90	1075

Table S14. Frequency of 248 families with poor, predicted, or high species richness based on strict consensus tabulated by primary realm presence. Statistics for a χ^2 independence test are presented, and categories in which more families were found for any of the three classes are presented in red, and those in which less families were found are in blue according to a Haberman residual analysis.

Realm	poor	predicted	high	Total
(χ^2 = 53.97, P < 0.0001)				
**Afrotropic**	18	11	0	29
**Australasia**	19	5	1	25
**Indomalayan**	2	5	0	7

**Nearctic**	4	7	0	11
**Neotropic**	22	84	6	112
**Oceania**	0	1	0	1
**Palearctic**	10	48	5	63
**Total**	75	161	12	248

Table S15. Frequency poor, predicted, and high species richness based on strict consensus tabulated by biome number. Statistics for a χ^2 independence test are presented, and categories in which more families were found for any of the three classes are presented in red, and those in which less families were found are in blue according to a Haberman residual analysis.

Biome	poor	predicted	high	Total
(χ^2 = 144.88, P < 0.0001)				
**1**	7	1	0	8
**2 to 4**	43	10	0	53
**5 to 7**	14	16	0	30
**8 to 10**	9	44	0	53
**11 to 13**	2	50	3	55
**14**	0	40	9	49
**Total**	75	161	12	248

Table S16. Frequency poor, predicted, and high species richness based on majority consensus tabulated by biome presence. Statistics for a χ^2 independence test are presented, and categories in which more families were found for any of the three classes are presented in red, and those in which less families were found are in blue according to a Haberman residual analysis.

Biome	poor	predicted	high	Total
(χ^2 = 58.45, P = 0.0003)				
**Boreal Forests/Taiga**	3	66	9	78
**Deserts & Xeric Shrublands**	30	147	12	189
**Flooded Grasslands & Savannas**	12	114	12	138
**Mangroves**	20	140	12	172
**Mediterranean Forests, Woodlands & Scrub**	23	106	11	140
**Montane Grasslands & Shrublands**	24	152	12	188
**Temperate Broadleaf & Mixed Forests**	36	119	12	167
**Temperate Conifer Forests**	18	113	12	143
**Temperate Grasslands, Savannas & Shrublands**	16	93	12	121
**Tropical & Subtropical Coniferous Forests**	16	136	12	164
**Tropical & Subtropical Dry Broadleaf Forests**	27	149	12	188
**Tropical & Subtropical Grasslands, Savannas & Shrublands**	35	149	12	196
**Tropical & Subtropical Moist Broadleaf Forests**	55	161	12	228
**Tundra**	2	49	10	61
**Total**	317	1694	162	2173

Table S17. Frequency poor, predicted, and high species richness based on strict consensus tabulated by primary biome presence. Statistics for a χ^2 independence test are presented, and categories in which more families were found for any of the three classes are presented in red, and those in which less families were found are in blue according to a Haberman residual analysis.

Biome	poor	predicted	high	Total
(χ^2 = 22.03, P = 0.6058)				
**Boreal Forests/Taiga**	1	0	0	1
**Deserts & Xeric Shrublands**	7	4	0	11
**Mangroves**	1		0	1
**Mediterranean Forests, Woodlands & Scrub**	7	13	1	21
**Montane Grasslands & Shrublands**	2	1	0	3
**Temperate Broadleaf & Mixed Forests**	18	34	5	57
**Temperate Conifer Forests**	0	3	0	3
**Temperate Grasslands, Savannas & Shrublands**	1	0	0	1
**Tropical & Subtropical Dry Broadleaf Forests**	1	2	0	3
**Tropical & Subtropical Grasslands, Savannas & Shrublands**	4	4	0	8
**Tropical & Subtropical Moist Broadleaf Forests**	33	100	6	139
**Total**	75	161	12	248

References

BELL, C. D., SOLTIS, D. E. & SOLTIS, P. S. 2010. The age and diversification of the angiosperms re-revisited. *American Journal of Botany,* 97**,** 1296-1303.

BOKMA, F. 2003. Testing for equal rates of cladogenesis in diverse taxa. *Evolution,* 57**,** 2469-2474, 6.

HERNÁNDEZ-HERNÁNDEZ, T. & WIENS, J. J. 2020. Why Are There So Many Flowering Plants? A Multiscale Analysis of Plant Diversification. *The American Naturalist,* 195**,** 948-963.

LI, H.-T., YI, T.-S., GAO, L.-M., MA, P.-F., ZHANG, T., YANG, J.-B., GITZENDANNER, M. A., FRITSCH, P. W., CAI, J., LUO, Y., WANG, H., VAN DER BANK, M., ZHANG, S.-D., WANG, Q.-F., WANG, J., ZHANG, Z.-R., FU, C.-N., YANG, J., HOLLINGSWORTH, P. M., CHASE, M. W., SOLTIS, D. E., SOLTIS, P. S. & LI, D.-Z. 2019. Origin of angiosperms and the puzzle of the Jurassic gap. *Nature Plants,* 5**,** 461-470.

RAMÍREZ-BARAHONA, S., SAUQUET, H. & MAGALLÓN, S. 2020. The delayed and geographically heterogeneous diversification of flowering plant families. *Nature Ecology & Evolution,* 4**,** 1232-1238.

REYES, E., SAUQUET, H. & NADOT, S. 2016. Perianth symmetry changed at least 199 times in angiosperm evolution. *TAXON,* 65**,** 945-964.

STEVENS, P. F. 2021. *Angiosperm phylogeny website, version 14* [Online]. Available: http://www.mobot.org/MOBOT/research/APweb/. [Accessed].

VAMOSI, J. C. & VAMOSI, S. M. 2011. Factors influencing diversification in angiosperms: At the crossroads of intrinsic and extrinsic traits. *American Journal of Botany,* 98**,** 460-471.

WIKSTRÖM, N., SAVOLAINEN, V. & CHASE, M. W. 2001. Evolution of the angiosperms: calibrating the family tree. *Proceedings of the Royal Society of London, Series B,* 268**,** 2211-2220.
