# Supplementary material for: Geographical and life-history traits associated with low and high species richness across angiosperm families
Source: Front Plant Sci. 2023 Nov 24;14:1276727. doi: 10.3389/fpls.2023.1276727 (PMC10722503; doi:10.3389/fpls.2023.1276727)
Supplement: Supplementary Data Sheet 3 — Supplementary Figures S1–S3 (.pdf). [file DataSheet_3.pdf]

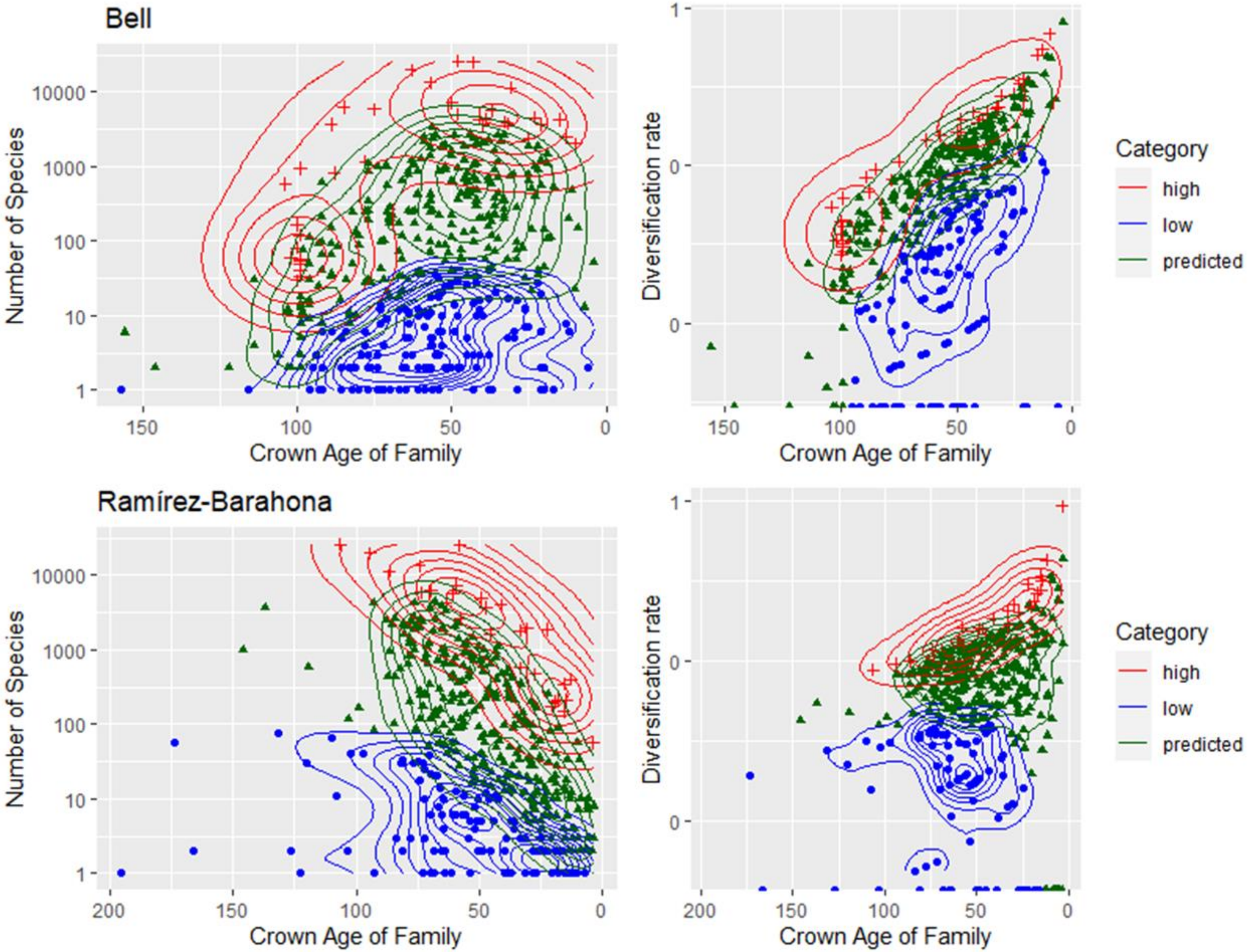

Supplementary Figure S1. Families categorized as having high, predicted or low SR (left panel) or DR (right panel) by family crown age based on the calibrated phylogenies from Bell (2010), and Ramirez-Barahona (2020).

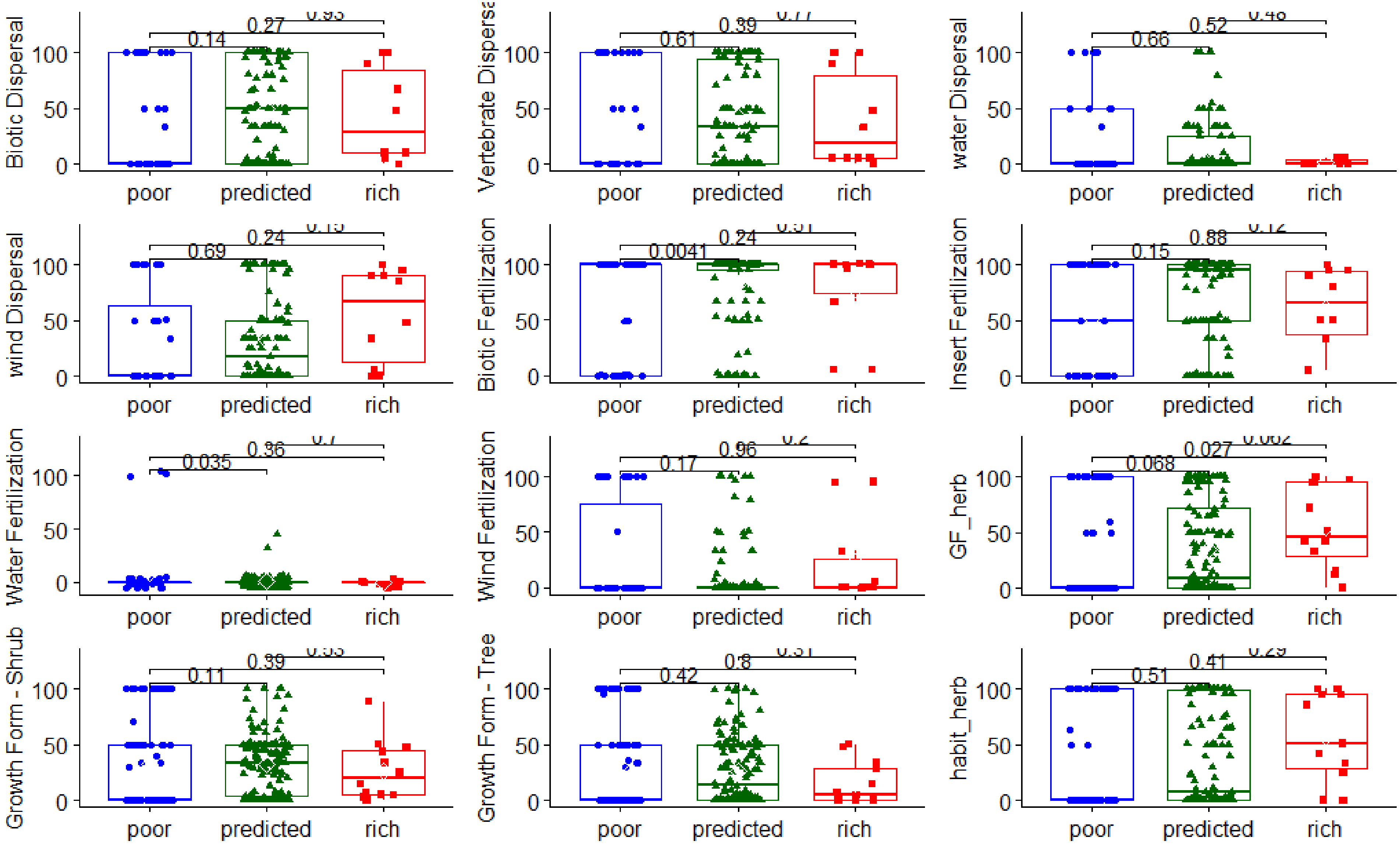

Supplementary Figure S2: Distribution of 12 traits across families with poor, predicted or high, species richness that showed a non-significant differences among SR categories, based on a Kruskal-Wallis, non-parametric rank test, at a global FDR = 0.1.

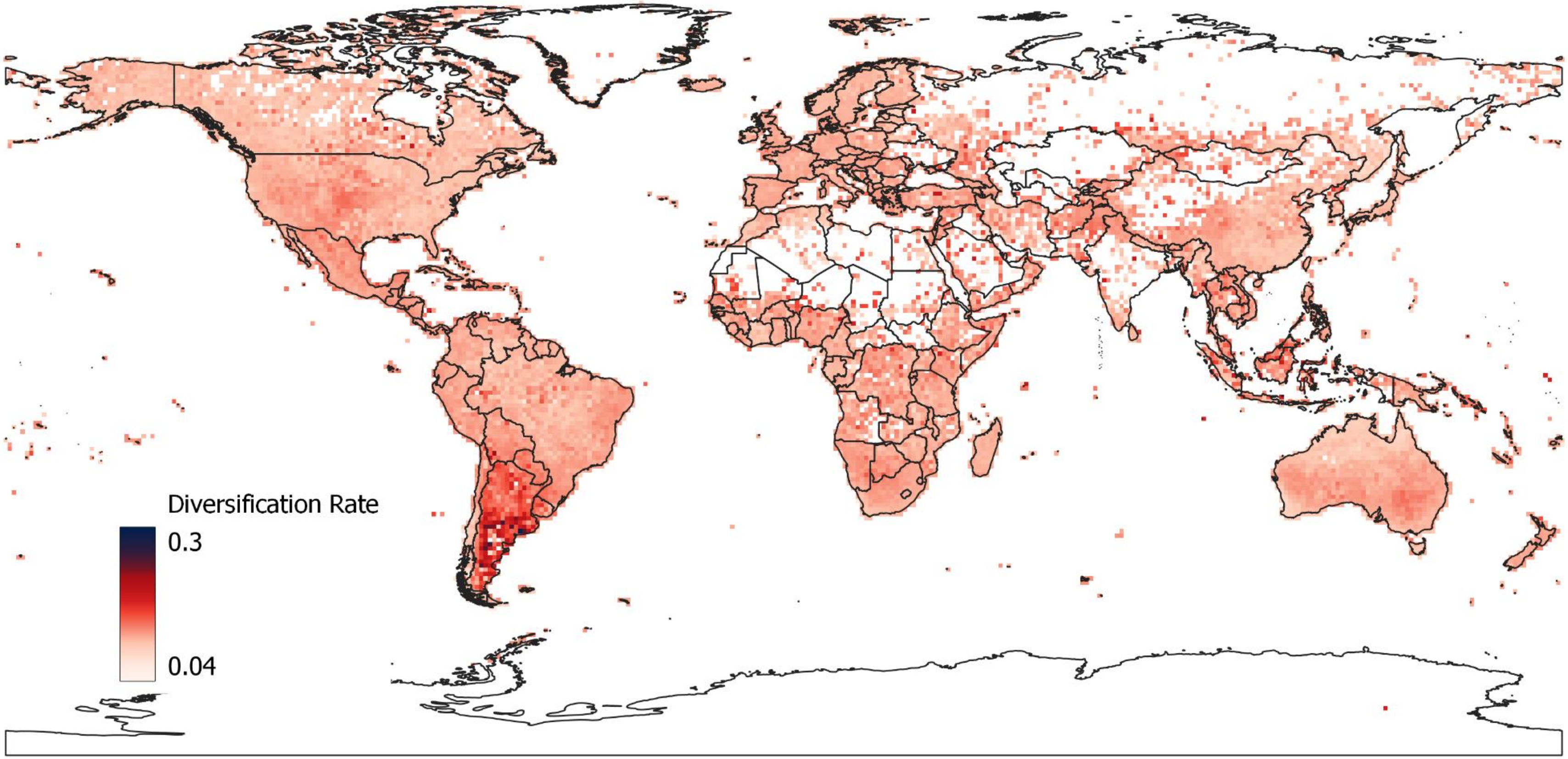

Supplementary Figure S3: mean diversification rate of families with predicted species richness in the globe (n.b. some regions are not well sampled). The colour of each pixel corresponds to the mean DR of the families to which a georeferenced data exists.

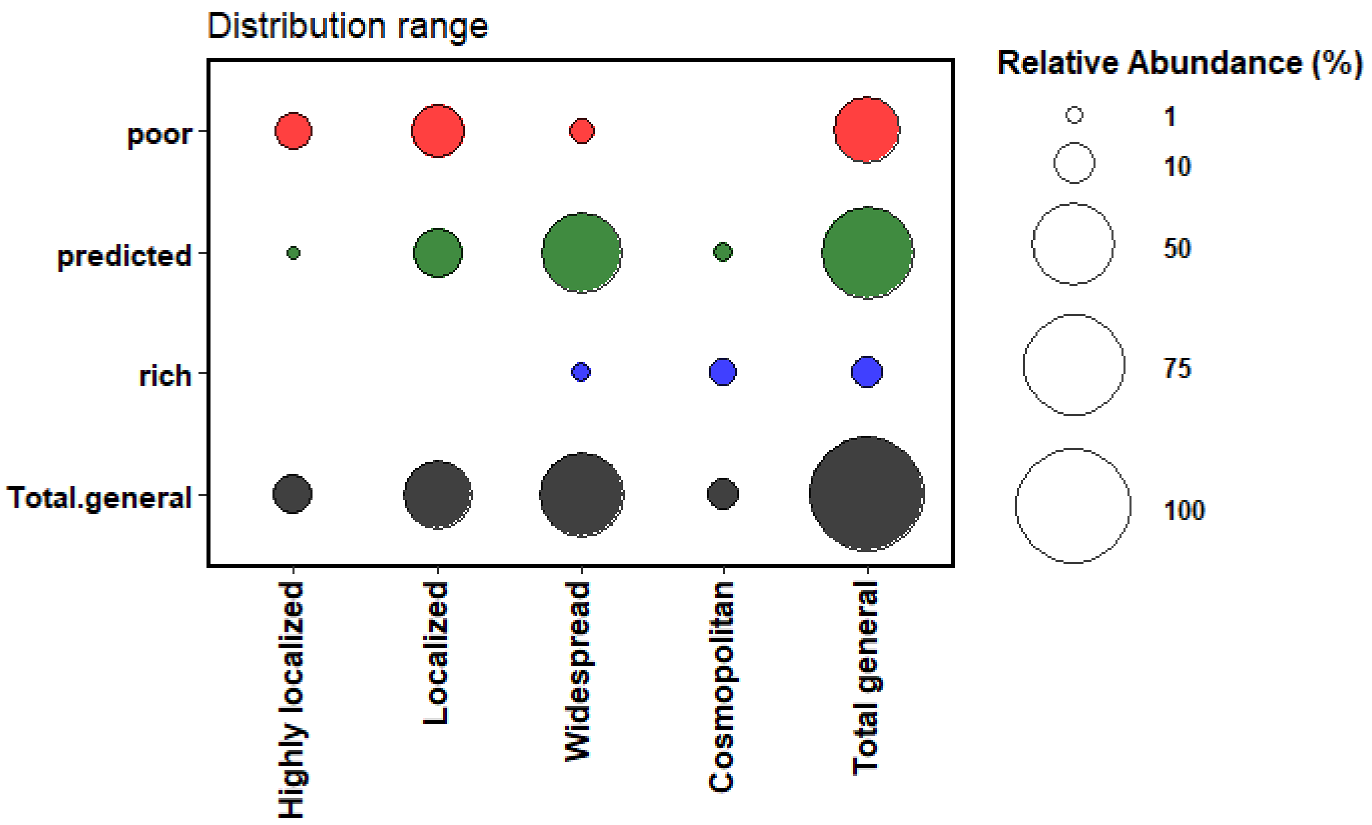

Supplementary Figure S4: Distributional range of families with poor, predicted or high SR

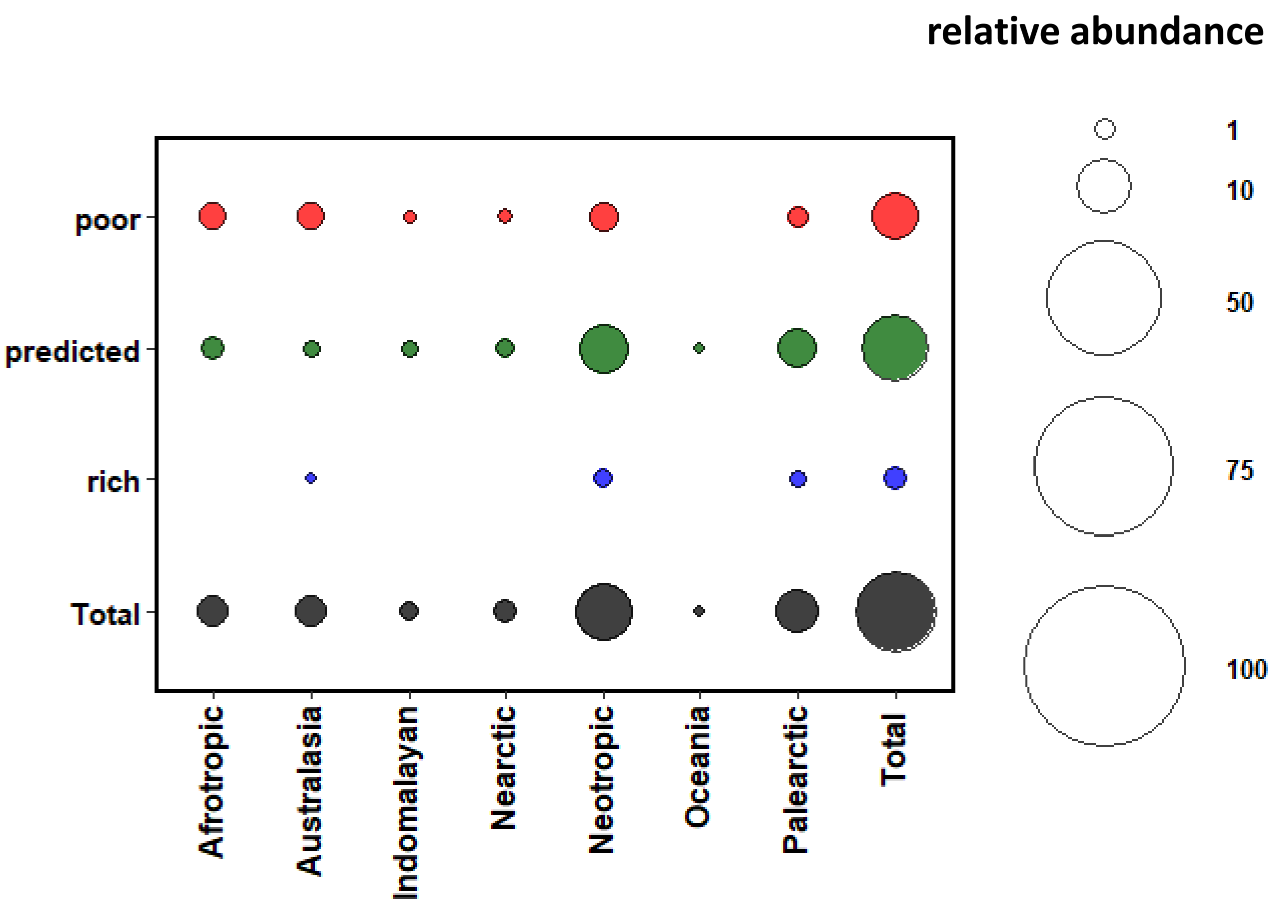

Supplementary Figure S5: Relative abundance in realms of families with poor, predicted or high SR
